# Biocompatible Electrospun Biomaterials for Advancing Thermoregulating Wearable Sensors in Next-Generation Smart Textiles

**DOI:** 10.3390/jfb17020100

**Published:** 2026-02-18

**Authors:** Sandra Varnaitė-Žuravliova, Žaneta Rukuižienė, Virginija Skurkytė-Papievienė, Paulė Bekampienė, Vykintė Trakšelytė, Julija Baltušnikaitė-Guzaitienė

**Affiliations:** 1 Department of Textile Technologies, Center for Physical Sciences and Technology, Demokratų str. 53, LT-48485 Kaunas, Lithuania; zaneta.rukuiziene@ftmc.lt (Ž.R.); virginija.skurkyte@ftmc.lt (V.S.-P.); paule.bekampiene@ftmc.lt (P.B.); vykinte.trakselyte@ftmc.lt (V.T.); julija.baltusnikaite@ftmc.lt (J.B.-G.); 2 Department of Production Engineering, Faculty of Mechanical Engineering and Design, Kaunas University of Technology, Studentų str. 56, LT-51424 Kaunas, Lithuania

**Keywords:** nanofibers, electrospinning, wearable-sensors, multifunctionality, biomaterials, biocompatibility, biosensor, thermoregulating, thermal properties, smart textiles

## Abstract

The rapid growth of electronic devices, including wearable sensors, has increased electronic waste, driving interest in sustainable, biocompatible materials. Electrospun biomaterials have emerged as versatile substrates for multifunctional wearable textiles, offering flexibility, high surface area, tunable porosity, and biocompatibility. Using natural polymers (e.g., silk fibroin, cellulose, chitosan) and synthetic polymers (e.g., polycaprolactone, polylactic acid, PVDF), electrospinning produces nanofibrous mats capable of supporting thermal regulation, moisture management, and integrated sensing for pressure, temperature, humidity, or chemical detection. Nature-inspired designs, hybrid composites, and advanced architectures enable passive and active thermoregulation via phase-change materials, thermochromic dyes, hydrogels, and conductive nanofibers, while maintaining wearer comfort, breathability, and skin safety. Despite progress, challenges persist in durability, washability, energy efficiency, manufacturing scalability, and recyclability. This review provides a comprehensive overview of biomaterials, fabrication techniques, multifunctional sensor integration, and thermoregulation strategies, highlighting opportunities for next-generation wearable textiles that combine sustainability, adaptive thermal management, and high-performance sensing.

## 1. Introduction

The convergence of advanced materials science and wearable electronics has catalyzed the emergence of smart textiles—fabrics embedded with functional components capable of sensing, responding, and adapting to environmental and physiological stimuli. Among the myriad of innovations driving this field, biocompatible electrospun biomaterials have garnered significant attention for their potential to serve as foundational substrates in wearable sensors, particularly those designed for thermoregulation.

**Figure 1:**
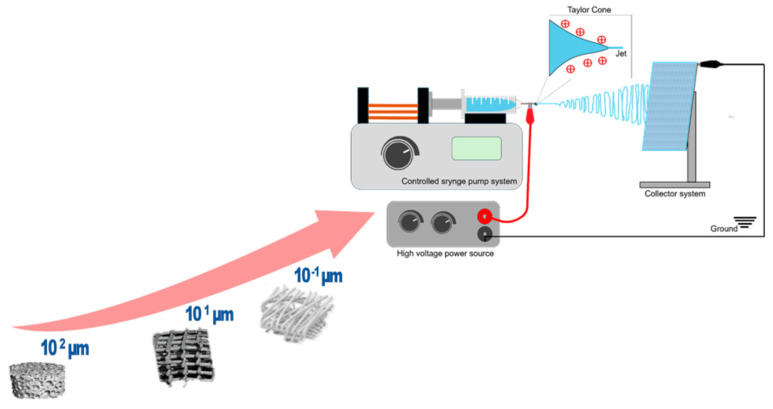
Illustration of electrospinning process (reprinted and adapted with permission under a Creative Commons license (CC BY 4.0) from Ref. [8]).

Electrospinning (see Figure 1), a versatile and scalable technique, enables the fabrication of nanofibrous mats with high surface area-to-volume ratios, tunable porosity, and mechanical flexibility—attributes that are critical for seamless integration into textiles and for maintaining wearer comfort [1]. When engineered from biocompatible polymers such as polyvinylidene fluoride (PVDF), silk fibroin, or polylactic acid (PLA), these electrospun fibers not only support physiological compatibility but also offer functional properties like piezoelectricity and thermal responsiveness [1,2,3,4,5,6,7].

The integration of such materials into wearable sensors has opened new avenues for real-time health monitoring, enabling the detection of vital signs such as skin temperature, hydration levels, and metabolic activity [9,10,11]. These sensors, when embedded in garments, can autonomously regulate thermal comfort by activating heating or cooling mechanisms in response to detected changes, thereby enhancing both performance and well-being in diverse environments [11,12,13].

Moreover, the shift toward sustainable and transient electronics has further underscored the importance of biodegradable and non-toxic materials in wearable systems. Electrospun biomaterials, particularly those derived from natural sources like silk fibroin, align with this vision by offering eco-friendly alternatives without compromising functionality [2].

The primary aim of this review is to provide a comprehensive overview of the current state, challenges, and future directions in the development of thermoregulating wearable sensors based on biocompatible electrospun biomaterials. As wearable technology becomes increasingly integrated into healthcare, sports, and personal wellness, the demand for materials that are both functional and skin-compatible has become paramount. Central to this discussion is the critical role of electrospinning technology in fabricating nanofibrous scaffolds from biocompatible polymers, which can be engineered to support thermal regulation in smart textiles. In this context, the review examines material selection, fabrication strategies, and sensor integration, while also addressing the challenges and opportunities that lie ahead in realizing next-generation smart textiles.

The novelty of this review lies in its integration of biocompatible electrospun biomaterials, thermoregulation, and multifunctional wearable sensors, demonstrating how advanced fiber architectures enable simultaneous passive and active thermal management, moisture control, and real-time sensing. It highlights sustainable material selection, hybrid composites, and eco-friendly phase-change systems, connecting material design to recyclability, energy efficiency, and environmental impact. By linking fabrication strategies, functional performance, and practical challenges, this work provides a comprehensive framework for guiding the development of next-generation, skin-friendly, and multifunctional smart textiles.

### 1.1. Rise of Smart Textiles and Wearable Sensors

The integration of electronics into textiles represents a pivotal advancement in the field of wearable technology, marking the emergence of smart fabrics—materials endowed with the capacity to sense, respond, and communicate with their surrounding environment. This progression reflects a broader trajectory in wearable systems, which have evolved from rudimentary fitness trackers into highly sophisticated, textile-embedded platforms capable of real-time physiological monitoring, environmental sensing, and adaptive functionality. Central to this transformation is the convergence of flexible electronics, nanotechnology, and advanced materials engineering, which collectively enable the seamless incorporation of sensors, actuators, and communication modules into fabric substrates. Consequently, garments are reconceptualized not as passive coverings but as dynamic interfaces that mediate interactions between the human body and its external milieu, with applications spanning health surveillance, environmental awareness, and individualized modes of expression. This paradigm shift underscores the interdisciplinary nature of smart textile research and highlights its potential to redefine both technological innovation and the cultural significance of clothing in contemporary society [12,13,14,15].

**Figure 2:**
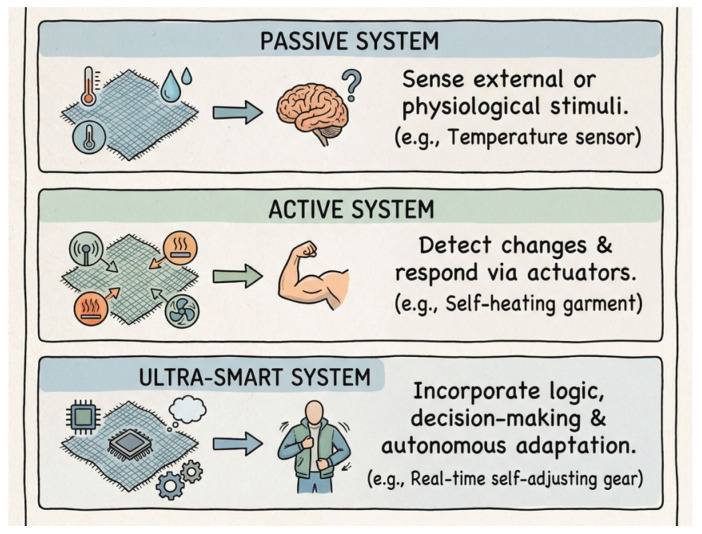
Classification of smart textiles (Image was generated using DeeVid AI Image Generator (DeeVid AI, 2026; https://deevid.ai/ai-image-generator, accessed on 3 February 2026)).

Smart textiles are generally classified into three categories: passive, active, and ultra-smart systems (see Figure 2). Passive textiles are capable of sensing external environmental or physiological stimuli, while active textiles not only detect changes but also respond through embedded actuators. Ultra-smart textiles extend these capabilities further by incorporating logic and decision-making functions, thereby enabling autonomous adaptation to dynamic conditions [14]. This classification reflects the progressive complexity and functionality of wearable systems, ranging from simple temperature sensors to garments that can adjust themselves in real time. Increasingly, these advanced functionalities are being realized through the integration of flexible sensors that monitor parameters such as temperature, strain, humidity, and biochemical markers, underscoring the interdisciplinary convergence of materials science, electronics, and nanotechnology in the development of next-generation wearable platforms.

The proliferation of wearable sensors, which serve as the cornerstone of smart textiles, is driven by significant advancements in flexible electronics, nanomaterials, and miniaturization. These developments have enabled the creation of sensors with enhanced sensitivity, biocompatibility, and adaptability, capable of monitoring a broad spectrum of physiological parameters—including heart rate, respiration, skin temperature, and hydration—without compromising user comfort or mobility. Progress in materials science, particularly the incorporation of conductive polymers, carbon-based nanomaterials, and electrospun nanofibers, has facilitated the fabrication of sensors that are lightweight, stretchable, and conformable to the skin. Moreover, the integration of conductive fibers and printed electronics allows sensors to be seamlessly embedded into textile substrates, thereby supporting continuous, non-invasive monitoring while preserving the breathability and wearability of conventional garments. Collectively, these innovations highlight the interdisciplinary convergence of electronics, nanotechnology, and materials engineering in advancing next-generation smart textile systems [15].

In healthcare, smart textiles are being used for remote patient monitoring, rehabilitation, and chronic disease management. In sports, they optimize performance and prevent injury by tracking biomechanical data. Military applications include uniforms that detect environmental hazards or monitor soldier vitals. Even fashion is embracing smart textiles for interactive and expressive clothing.

The market for smart textiles is expanding rapidly. According to Grand View Research, it is projected to exceed $12 billion by 2025, fueled by consumer demand, technological innovation, and cross-sector adoption [16]. However, challenges remain in durability, washability, energy autonomy, and data privacy. Researchers are exploring solutions such as energy-harvesting fabrics, biodegradable electronics, and secure data protocols to address these concerns.

As the field evolves, biocompatible electrospun biomaterials are emerging as a promising foundation for wearable sensors. Their flexibility, breathability, and compatibility with human skin make them ideal for thermoregulating applications—ensuring comfort while enabling precise temperature control in dynamic environments.

### 1.2. Thermal Regulation as a Core Challenge in Long-Term Wearable Comfort

As wearable technologies become increasingly integrated into daily life—from fitness trackers and smart clothing to medical monitoring systems—the issue of thermal regulation has emerged as a central challenge in ensuring sustained user comfort and device performance. Unlike conventional electronics, wearables are in direct contact with the skin, making heat management essential not only for operational stability but also for preventing discomfort, irritation, or even thermal injury.

Human skin is highly sensitive to temperature fluctuations, and prolonged exposure to elevated temperatures from embedded electronics can lead to sweating, skin fatigue, and reduced adherence to the device. Conversely, inadequate warmth in cold environments can compromise sensor accuracy and user experience. Therefore, maintaining a stable microclimate between the skin and the wearable interface is vital for long-term usability.

**Figure 3:**
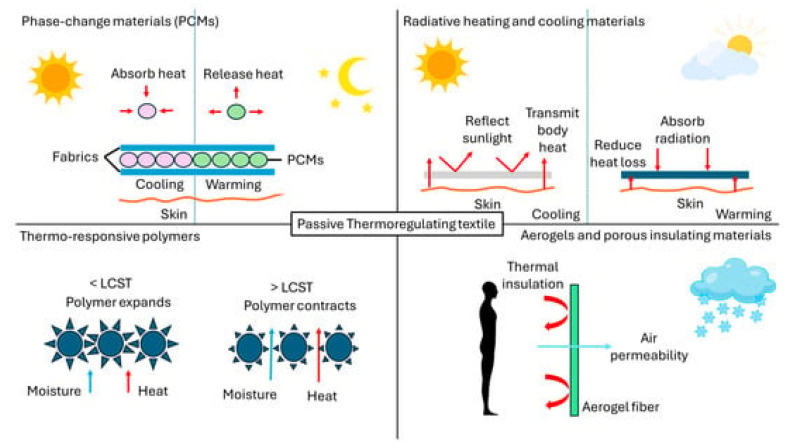
Passive thermoregulating textiles (reprinted and adapted with permission under a Creative Commons license (CC BY 4.0) from Ref. [18]).

Recent advances in thermoregulating textiles have focused on both passive and active strategies. Passive approaches include the use of phase change materials (PCMs), reflective coatings, and porous structures that facilitate heat dissipation. The types of passive thermoregulating textiles are presented in Figure 3. Active systems incorporate thermoelectric modules, microfluidic cooling, and responsive polymers that adapt to temperature changes in real time [17,18].

Electrospun nanofibers have shown promise in this domain due to their high surface area, tunable porosity, and ability to incorporate functional additives. For instance, integrating carbon-based nanomaterials or metallic nanoparticles into electrospun mats can enhance thermal conductivity, enabling efficient heat transfer away from the skin [11,12,13,19]. Moreover, biocompatible polymers such as silk fibroin and polycaprolactone (PCL) offer breathable and skin-friendly platforms for sensor integration.

Despite these innovations, several challenges persist. Durability under repeated thermal cycles, washability, and energy efficiency remain key concerns. Additionally, balancing thermal regulation with other performance metrics—such as sensor sensitivity, mechanical flexibility, and aesthetic appeal—requires multidisciplinary design strategies [19].

Addressing these challenges is crucial for the next generation of smart textiles, particularly in healthcare, sports, and military applications where long-term wear is common. Future research must focus on hybrid systems that combine passive and active thermal management, sustainable materials, and intelligent feedback mechanisms to optimize comfort and performance [12,19].

### 1.3. The Role of Biocompatible Materials and Electrospinning Technology

The development of wearable sensors for smart textiles hinges on the use of materials that are both functional and safe for prolonged skin contact. Biocompatible polymers—such as polycaprolactone (PCL), polylactic acid (PLA), silk fibroin, and chitosan—have emerged as ideal candidates due to their non-toxic, biodegradable, and skin-friendly properties [2,4,5,6,7,8,9]. These materials offer mechanical flexibility, breathability, and compatibility with human tissue, making them suitable for long-term wearable applications.

Electrospinning technology plays a pivotal role in transforming these polymers into nanofibrous mats with high surface area, tunable porosity, and excellent mechanical compliance. This technique uses electrostatic forces to draw polymer solutions into ultrafine fibers, producing structures that mimic the extracellular matrix and conform well to the skin [1]. The resulting mats are highly breathable and can be engineered to incorporate functional additives such as conductive nanoparticles, phase change materials, or antimicrobial agents, enabling multifunctional capabilities in smart textiles.

For example, silk fibroin electrospun with carbon quantum dots has demonstrated promise in transient electronics due to its biodegradability and thermal responsiveness [2]. Similarly, electrospun PVDF and its copolymers have been explored for their piezoelectric properties, enabling self-powered sensing platforms in wearable systems [1].

Despite these advances, challenges remain in achieving consistent fiber morphology, mechanical robustness, and scalable manufacturing. Researchers are actively exploring hybrid materials that combine natural and synthetic polymers, as well as green solvents and integrated wireless modules, to create fully autonomous and sustainable smart textile systems [11,12,13].

## 2. Biocompatibility in Thermoregulating Wearable Sensors

The integration of thermoregulating sensors into wearable textiles involves continuous and intimate contact with human skin, often under dynamic conditions such as motion, perspiration, and fluctuating temperatures. In this context, biocompatibility becomes a functional necessity, not only to ensure skin safety, but also to maintain comfort and long-term usability of the device. While thermoregulating smart textiles are typically not classified as medical devices, they are nonetheless expected to comply with high standards of skin compatibility, particularly when intended for extended or repeated wear.

In wearable thermoregulating sensors, biocompatibility extends beyond the mere absence of toxicity and encompasses a range of properties that contribute to skin comfort, biological inertness, and mechanical harmony with the body. These include non-irritating surface chemistry, breathability and effective moisture management, mechanical softness and flexibility, and resistance to microbial growth in humid, thermally active environments. Unlike implantable materials, which must be evaluated for systemic biological effects, wearable biomaterials primarily emphasize epidermal safety and comfort during motion and perspiration.

**Figure 4:**
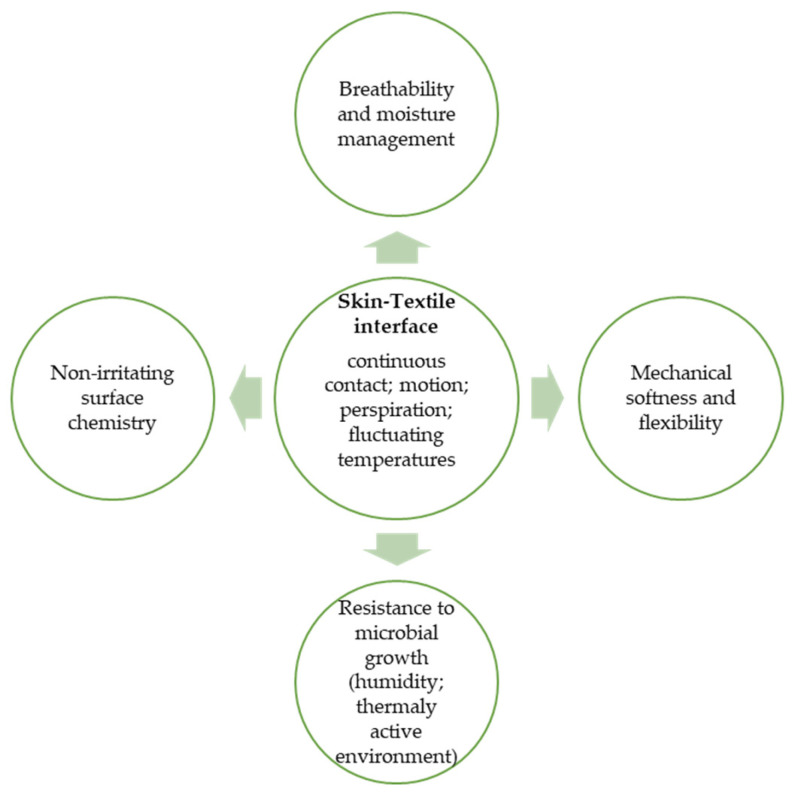
Conceptual framework for skin-textile interface biocompatibility.

To clarify the relevance of these factors in thermoregulating garments, the following section summarizes how material architecture governs moisture and heat at the skin-textile interface. Figure 4 schematically illustrates the system-level factors influencing biocompatibility in thermoregulating wearable sensors, as discussed in the subsequent sections.

In this context, biocompatibility should be interpreted not as a static material property, but as a dynamic system-level response shaped by skin–textile interactions under real-use conditions. In addressing continuous skin contact under dynamic conditions, various studies have highlighted the importance of material design in enhancing moisture management and reducing skin irritation. Zhao et al. [20] demonstrated that manipulating the architectural (via fluorine-free waterborne coatings on fibrous substrates) properties of fabrics can significantly improve moisture-vapor transmission, effectively keeping liquids away from the skin and thus mitigating the risk of irritation; this aligns with findings from Park et al. [21], who evaluated moisture management in high-temperature-resistant nanofibrous membranes and underscored the coupling between breathability and thermal protection. These observations are consistent with Troynikov and Wardiningsih [22], who examined active garments and found that fabric architecture governs the stability of the skin microclimate during exertion, emphasizing how textile mechanics and porosity steer sweat management and comfort in practice. Taken together, these results set up the link between microstructure, microclimate, and perceived comfort during exertion. Taken together, these studies indicate that control of the skin microclimate through textile architecture is a primary driver of biocompatibility in thermoregulating wearables, rather than a secondary comfort-enhancing feature.

Expanding on the relationship between comfort and functionality, Xie et al. [23] and Zhang et al. [24] indicated that carefully tailoring the properties of textile materials strikes a balance between breathability and water resistance, thereby facilitating both comfort and performance during prolonged wear. Wang et al. [25] further illustrated the application of these principles in healthcare electronics by demonstrating how MXene-based, porous, and breathable materials can maintain optimal sensing performance and antimicrobial properties even during motion, thereby enhancing both user comfort and device functionality. The versatility of these materials reflects the growing trend toward integrating comfort with technological performance in wearable health monitoring solutions. This convergence suggests a broader shift in wearable design philosophy, where biocompatibility and sensing performance are commonly co-optimized rather than treated as competing objectives.

Mechanical harmony between wearable devices and the human body represents a second, equally critical dimension of biocompatibility, and emerges across studies as a limiting factor for long-term comfort and signal fidelity during motion. A consistent conclusion across studies is that mechanical mismatch, rather than chemical incompatibility alone, often underlies discomfort, irritation, and signal degradation during motion. Reviews by Xie et al. [23] and Khan et al. [26] emphasize that co-engineering biocompatibility and mechanical compliance within polymer and textile substrates is essential for achieving stable on-skin operation.

Recent material strategies increasingly demonstrate that enhanced functionality in thermoregulating wearables does not need to compromise biocompatibility, but instead, it can reinforce comfort and long-term usability. Peng et al. [27] reported the fabrication of breathable, biodegradable, and antibacterial electronic skins that improve compatibility and comfort during operation. Wu et al. [28] expanded on this concept through the development of permeable electrodes designed to form conformal interfaces with skin, ensuring sustained comfort and functionality even during vigorous activities. Similarly, Liu et al. [29] showed that polyvinylidene fluoride-based membranes exhibit ultra-flexibility, strong waterproofing, and breathability, which are key attributes for electronic skin applications, highlighting the importance of integrating functional materials in wearable technology.

These advancements exemplify the potential for smart textiles to improve the interface between the wearer and the garment, promoting both health and comfort. Further enhancing functionality at the skin-textile interface, Szewczyk et al. [30] demonstrated that oil-infused polymer fiber membranes can improve skin hydration, a critical factor for maintaining skin health during extended wear. In line with this, permeable triboelectric fiber mats designed by Maksoud et al. [31] exhibit mechanical properties closely matched to those of human skin, maintaining comfort and performance output even under intense motion.

Collectively, these developments underscore the profound impact of advanced textile engineering on wearable technology and user experience. This body of work reinforces the view that material architecture enables simultaneous gains in comfort, durability, and functional performance during long-term wear.

The integration of advanced textile design and material properties is crucial for maximizing comfort and functionality in wearable technologies. Continuous innovation in this field is essential for developing effective solutions that prioritize the wearer’s experience, particularly in active and healthcare applications. Importantly, this body of evidence indicates that material and structural choices at the textile level increasingly dictate system-level performance and user acceptance in thermoregulating wearables.

Beyond individual material demonstrations, multiple studies indicate that mechanical softness, stretchability, and elastic recovery are decisive factors for long-term wearability. McLaren et al. [32] identified compliant sensor placement and deformable designs as key factors in improving user experience in neurological rehabilitation textiles, while analyses of wearable ECG systems [33] have linked mechanical compliance directly to reduced motion artifacts and improved patient safety. Stretchable and self-adhesive electrodes developed by Ding et al. in [34], along with survey-based evidence from Yin et al. in [35], further confirm that elasticity and shape recovery mitigate irritation during active use. Additional studies [36,37,38,39,40] reinforce the conclusion that soft mechanics enhance adhesion, reduce friction, and preserve sensing performance, positioning mechanical biocompatibility as a prerequisite for reliable thermoregulating wearables rather than an auxiliary comfort feature. Accordingly, mechanical biocompatibility should be regarded as a prerequisite for reliable thermoregulating wearables, rather than merely an auxiliary comfort consideration.

In exploring the critical aspect of regulatory alignment regarding biocompatibility in thermoregulating wearable sensors, it is vital to underscore the importance of adherence to established safety standards, particularly in the context of new material formulations and their interactions with biological systems. Patel et al. in [41] and Sharma et al. in [42] highlighted the imperative for rigorous safety evaluations aligned with ISO 10993 standards, which address aspects such as cytotoxicity (ISO 10993-5 [43]) and irritation testing on reconstructed human epidermis (ISO 10993-23 [44]) [45]. This shift towards continuous on-body monitoring necessitates that all materials used in wearable technologies undergo thorough assessment of their effects on human tissues to ensure long-term usability without adverse reactions. European regulatory adoption has reinforced this shift, with EN ISO 10993-23:2021/A1:2025 [46] clarifying RhE endpoints and moving decisively away from legacy animal models.

Advancing this discussion, Liu et al. in [47] examined the significance of biocompatibility for wearable sensors crafted from innovative materials such as self-healing hydrogels, emphasizing that the effectiveness of these sensors depends heavily on maintaining skin-friendly interfaces while delivering consistently high performance across variable environments. Similar concerns were echoed by Ereifej et al. in [48], who noted that cytotoxicity testing serves as a preliminary step for evaluating the compatibility of various biomaterials in medical devices, thereby establishing a foundation for their safe application. These regulatory frameworks collectively reposition biocompatibility from a material-level checklist to a system-level validation process aligned with continuous, on-body use. As such, regulatory alignment functions not only as a compliance requirement but also as a design constraint that shapes material selection and device architecture from early stages of development.

Moreover, Choi et al. in [49] underscored the need for biocompatibility assessments in wearable sensors employing ionic liquids, advocating in vitro testing on human keratinocyte and fibroblast cells to ensure that these materials do not elicit toxic responses. Such regulatory scrutiny is echoed by other research, which has demonstrated that the use of biocompatible materials, particularly naturally derived substrates such as silk fibroin, facilitates the development of soft, skin-tolerant textile sensors that comply with ISO testing protocols [50]. Wang et al. in [51] further linked material stability in health-monitoring applications to consistent performance at the skin interface.

Antimicrobial performance has been investigated as a co-requirement in humid microclimates, where microbial growth can pose significant challenges. Windmiller and Wang in [52] reviewed on-body chemical and biochemical sensing and underscored that antimicrobial nanomaterial must be integrated with careful consideration of comfort and safety. Baldo et al. in [53] surveyed biodegradable and transient sensors and concluded that natural or hydrolysable matrices can reduce long-term bioburden. Sen et al. in [54] discussed antimicrobial electrospun fibers for durable, long-contact applications, while Shafique et al. in [55] analyzed hydrogel-based sensors and highlighted low cytotoxicity and skin comfort under moisture-rich conditions. Krysiak et al. in [56] examined antimicrobial treatments in flexible textiles, explicitly linking hygiene performance with mechanical comfort.

Liakos et al. in [57] tested cellulose acetate electrospun pads loaded with essential oils and demonstrated suppression of common pathogens without sacrificing breathability. Yin et al. in [58] presented silk-sheathed conductive wires that provided splash resistance and electrical insulation in a skin-friendly, washable format. Across these studies, authors consistently identified ISO 20743 [59] (along with AATCC 100 [60] where applicable) as the appropriate, textile-specific standard for evaluating antibacterial efficacy. This consensus emphasizes that antimicrobial performance must be assessed within textile-relevant testing frameworks to meaningfully support long-term skin compatibility.

Finally, to close the safety loop beyond comfort and hygiene, sensitization and chemical compatibility have been framed by researchers as critical to long-term wear. Iadaresta et al. in [61] showed that textile-related chemicals such as benzothiazole can migrate to the skin under wear-like conditions, reinforcing the need for chemical safety and low-irritant formulations. He et al. in [62] developed integrated textile sensor patches based on silk-derived carbon textiles and demonstrated non-invasive measurement using skin-tolerant materials, underscoring that device-level chemistry and surface finishes must meet dermatological safety expectations. Armengol et al. in [63] discussed allergenic risks associated with textile finishes and emphasized the importance of regulating dyes, crosslinkers, and auxiliary agents to minimize the incidence of allergic contact dermatitis in both medical and consumer textiles.

In the regulatory context, the harmonized standard EN 1811:2023 [64] under REACH [65] sets the nickel-release test method and compliance decision limit (≤0.88 µg·cm^−2^·week^−1^) for metal components in prolonged skin contact, thereby guiding the selection of snaps, connectors, and electrodes in wearable systems. Wang et al. in [66] reviewed surface-engineered biomaterials for wound management and demonstrated that benign coatings and passivation strategies can effectively reduce sensitization risks, offering translatable approaches to wearable biosensors. In parallel, Wang et al. in [67] explored biodegradable polysaccharide matrices for humidity sensing as a route to achieving function performance using inherently low-irritant chemistries.

Zeybek, B. & Duman, M. in [68] examined electrospun sensing platforms and emphasized the importance of chemical stability and biocompatibility in minimizing dermal irritation. Similarly, Liu et al. in [69] and Tang et al. in [70] investigated tunable composite nanogenerators and reinforced that active-layer engineering must account for skin-exposure chemistry from the earliest stages of design. Collectively, these findings emphasize that chemical stability and sensitization control are integral components of biocompatibility, particularly for thermoregulating wearables intended for prolonged skin contact.

Altogether, the literature converges on a practical, evidence-based definition of biocompatibility for thermoregulating textile wearables. This definition encompasses electrospun, breathable, and mechanically compliant architectures that preserve the skin microclimate and mechanical comfort; material chemistries validated through ISO 10993-aligned cytotoxicity and irritation assessments, alongside textile-specific antimicrobial testing (ISO 20743 [59]); and article-level controls on sensitizers and metallic components through REACH [65] and EN 1811 [64], with RoHS [71] constraints applied where electronic elements are involved. Within this framework, thermoregulation, comfort and safety are no longer competing objectives but instead emerge as co-designed features of next-generation smart textiles.

## 3. Electrospinning of Biomaterials for Thermoregulating Textile Interfaces

### 3.1. Principles and Advances in Electrospinning for Functional Fiber Fabrication

The principle of electrospinning technology lies in the ability of a conductive polymer, either in solution or melt form, to generate continuous fibrous structures under a high-voltage electric field through elongation between a spinning electrode and a collector. For laboratory-scale polymer trials, a single-tip or single-nozzle configuration is commonly employed, where a polymer droplet is placed onto the spinning electrode [72,73]. Upon application of an electric field, the droplet deforms into a conical structure known as a Taylor cone, from which a fine polymer jet is ejected once the applied voltage exceeds the threshold required to overcome the surface tension of the liquid polymer [74]. Studies demonstrate that this versatile setup can be effectively used to fabricate functional material prototypes for wearable sensor and textile applications [72,73]. When polymer feeding is continuous, uninterrupted fiber production can be achieved, enabling scalability toward industrial manufacturing [75].

Beyond sensing performance, thermoregulation is a critical function of wearable textile interfaces, as maintaining skin temperature within a comfortable range directly influences user comfort, physiological performance, and long-term wearability. Electrospinning is particularly well suited for thermoregulating textiles due to its ability to produce highly porous, lightweight, and breathable fibrous membranes with precisely tunable morphology. The inherently high surface-area-to-volume ratio of electrospun nanofibers promotes efficient heat dissipation and moisture evaporation, which are essential mechanisms for passive cooling. By controlling fiber diameter, porosity, alignment, and layer thickness, electrospun mats can be engineered to serve either as thermal insulation layers, by trapping air within the fibrous structure, or as cooling layers that enhance sweat evaporation and convective heat transfer at the skin-textile interface.

**Figure 5:**
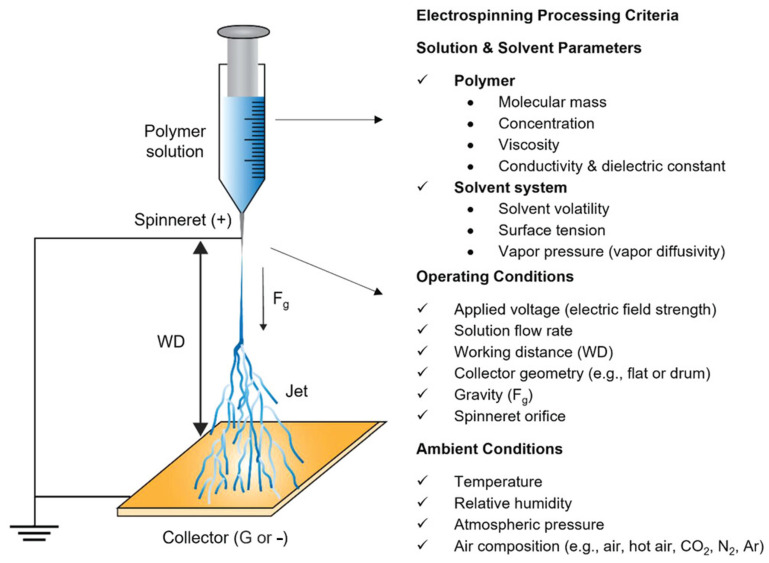
Schematic representation of the electrospinning processing parameters (reprinted and adapted with permission under a Creative Commons license (CC BY 4.0) from Ref. [76]).

The most commonly employed electrospinning techniques are solution electrospinning and melt electrospinning, each offering distinct advantages and limitations depending on the intended application. Solution electrospinning involves dissolving a polymer in a suitable solvent to form a spinnable solution. Under a high-voltage electric field, a charged jet is ejected from the needle tip, and as the jet travels toward the collector, the solvent evaporates, leaving behind a solidified fiber [76]. Solvent evaporation drives significant jet thinning, enabling the formation of ultrafine fibers with diameters from a few tens of nanometers to several micrometers, which makes the technique well suited for applications that demand high surface area, fine porosity, and delicate structural features. Achieving such fiber quality requires multiple factors to act simultaneously under favorable conditions, including parameters related to the solution, the operating setup, and the surrounding environment. These interconnected conditions ultimately govern the process’s production rate and shape the physicochemical and morphological characteristics of the resulting materials [76]. The schematic representation of the electrospinning processing parameters are presented in Figure 5.

A major advantage of solution electrospinning in the context of wearable sensors and thermoregulating textiles is its broad material compatibility. A wide range of polymers, including biopolymers, conductive polymers, carbon-based nanomaterial composites, and stimuli-responsive materials, can be electrospun from solution [77]. This versatility enables the fabrication of fibers with precisely tailored electrical, thermal, and mechanical properties. Additionally, because solution electrospinning typically operates at ambient or moderately elevated temperatures, it is well suited for incorporating thermally sensitive bioactive molecules, such as enzymes, antibodies, growth factors, or even living cells, without compromising their structural integrity or biological function [77,78]. This capability is particularly valuable for next-generation smart textiles that integrate biosensing or therapeutic functionalities.

However, solution electrospinning also presents challenges. The use of volatile, flammable, or toxic solvents raises environmental, safety, and regulatory concerns, particularly for wearable applications where fibers come into direct contact with the skin [79]. Residual solvent trapped within fibers can compromise mechanical performance, biocompatibility, and long-term stability, making complete solvent removal essential. Furthermore, solvent evaporation rates strongly influence fiber morphology, potentially leading to defects such as beads, pores, or inconsistent diameters if processing conditions are not carefully controlled.

In contrast, melt electrospinning eliminates the need for solvents entirely. In this approach, the polymer is heated above its melting temperature to form a viscous melt, which is then electrospun under a high-voltage field. As the molten jet travels toward the collector, it solidifies through cooling, forming continuous fibers [80]. Because no solvent evaporation occurs, the jet experiences less thinning, resulting in fibers that are generally thicker—often in the micrometer range—compared to solution-spun fibers [81]. While this may limit applications requiring nanoscale features, it provides advantages for structural components where mechanical robustness is essential.

Melt electrospinning is inherently more environmentally friendly, as it avoids solvent emissions and reduces the need for post-processing purification. It is also well suited for large-scale industrial production, particularly when combined with techniques such as melt electrowriting, which enables precise fiber placement. The high polymer concentration in the melt contributes to enhanced mechanical strength, making melt-spun fibers attractive for durable textile interfaces, especially those intended for repeated washing, stretching, or mechanical stress.

**Figure 6:**
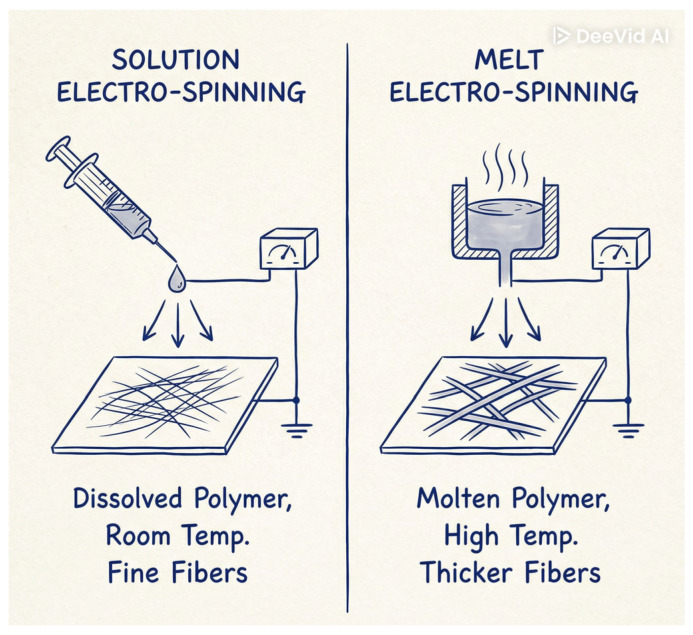
The simplified comparison of solution electrospinning and melt-spinning processes (Image was generated using DeeVid AI Image Generator (DeeVid AI, 2026; https://deevid.ai/ai-image-generator, accessed on 3 February 2026)).

Nevertheless, melt electrospinning has its own limitations. Only thermoplastic, thermally stable polymers can be processed, which restricts material selection [82]. High processing temperatures may degrade sensitive additives, preventing the incorporation of bioactive molecules or certain conductive fillers. Additionally, heating polymers to their melting point results in higher energy consumption, which can increase production costs. The higher viscosity of polymer melts also makes it more challenging to achieve very fine fiber diameters, limiting the achievable porosity and surface area compared to solution electrospinning. A comparison of melt electrospinning and solution electrospinning is presented in Table 1. The simplified comparison of solution electrospinning and melt-spinning is presented in Figure 6.

Traditional electrospinning provides limited control over fiber alignment, patterning, and functional anisotropy, which are important parameters for both sensing accuracy and thermal management. To overcome these limitations, various electrospinning process modifications (e.g., magnetic-field or airflow assistance) and hardware adaptations (e.g., spinneret or collector design) have been developed. For example, magnetic field-assisted electrospinning enables control over jet trajectory through the incorporation of magnetic nanoparticles (e.g., Fe_3_O_4_, Fe_2_O_3_, CoFe_2_O_4_) into polymer solutions [83] or by using magnetically patterned collectors [84]. These approaches yield highly aligned nanofibers with improved electrical conductivity and mechanical responsiveness, which can also facilitate directional heat transport within textile structures [85]. Airflow-assisted electrospinning introduces additional aerodynamic forces through pressurized air streams during coaxial or side-by-side spinning. This modification influences jet stretching, drying rate, and fiber morphology, allowing enhanced control over porosity and thickness—key parameters for regulating heat and moisture transport in wearable textiles [85]. For applications requiring high deposition precision, such as patterned heating or cooling zones, techniques like near-field electrospinning and melt electrowriting are employed, in which the spinneret-to-collector distance is reduced to the sub-millimeter to centimeter range to achieve accurate fiber placement [86,87,88].

Spinneret design plays a critical role in defining fiber morphology, porosity, and functional integration. Spinnerets used for wearable sensor and thermoregulating textile fabrication range from needleless (free-surface) systems [89] to single, double (coaxial or side-by-side) [90], and multifluid spinnerets [79]. Needleless electrospinning, which utilizes rotating drums or discs as spinning sources [91], offers high production rates but provides limited control over fiber uniformity and alignment. In contrast, multifluid spinnerets offer enhanced fluid dynamic control and enable advanced fiber architectures. Concentric (uniaxial) spinnerets facilitate core–shell fiber formation, allowing encapsulation of sensitive biomolecules or conductive fillers (e.g., PEDOT: PSS), while parallel or side-by-side configurations produce Janus fibers with spatially distinct functionalities [92]. Such architectures are particularly valuable for thermoregulating textiles, where different fiber domains can independently manage moisture transport, thermal insulation, or sensing.

Finally, collector geometry significantly influences fiber organization. Flat collectors typically produce randomly oriented fiber mats, while rotating or cylindrical collectors enable semi-aligned to highly aligned structures [93]. Fiber alignment not only improves mechanical and electrical properties but also affects directional heat transfer and airflow through the textile, further enhancing thermoregulating performance.

### 3.2. Electrospun Biomaterial Design: Polymers, Solvents, Additives, and Functionalization Strategies

Material selection plays a decisive role in determining both the sensing and thermoregulating performance of electrospun wearable textile interfaces. Electrospinning relies on a delicate balance between electrostatic forces, surface tension, and viscosity of the fiber-forming polymer and, in the case of solution electrospinning, the solvent system. Beyond spinnability, the choice of polymers, solvents, and additives directly influences fiber morphology, porosity, wettability, electrical conductivity, mechanical flexibility, moisture transport, and thermal behavior. Therefore, the primary criteria for material and additive selection include biocompatibility, biodegradability, functional response, and long-term stability during skin contact.

The main fiber-forming biomaterials used in electrospun wearable systems can be broadly classified into natural, synthetic, and composite polymers. Natural biopolymers derived from biological sources—such as proteins (collagen, gelatin, silk fibroin, elastin) and polysaccharides (chitosan, alginate, hyaluronic acid, starch, cellulose and its derivatives)—are inherently biocompatible and biodegradable. Many of these materials exhibit favorable hygroscopicity, moisture absorption, and breathability, making them particularly suitable for skin-contact layers that promote evaporative cooling and thermal comfort.

Synthetic polymers, including polycaprolactone (PCL), polylactic acid (PLA), polyglycolic acid (PGA), poly(lactic-co-glycolic acid) (PLGA), polyurethane (PU), and polyethylene oxide (PEO), offer superior mechanical durability, elasticity, and processability. Their tunable mechanical properties enable integration into stretchable textile substrates while maintaining structural integrity under repeated deformation. Blending natural and synthetic polymers is a widely adopted strategy to simultaneously optimize thermal insulation, moisture management, sensing performance, and mechanical robustness, which is essential for thermoregulating textile interfaces exposed to dynamic environmental and physiological conditions.

Solvent selection is a critical factor for biomaterial applications in wearable sensors and thermoregulating textiles, as it directly influences polymer compatibility, toxicity, and solution processability, with key parameters including volatility, conductivity, and viscosity. Typical solvents for various fiber-forming polymers are summarized in Table 2.

Certain acids, such as acetic acid, formic acid, and lactic acid, can serve dual roles as both solvents and functional additives, modifying solution conductivity, viscosity, and pH to enable finer fibers with increased porosity. Such morphological control enhances breathability, moisture transport, and cooling efficiency, which are essential for thermoregulating textile interfaces. The choice of polymer-solvent systems also governs fiber morphology, porosity, mechanical performance, and functional integration in electrospun textiles. Biopolymers offer excellent biocompatibility, moisture management, and eco-friendliness, but often rely on potentially hazardous solvents that complicate large-scale, safe production. In contrast, synthetic polymers provide mechanical robustness, thermal stability, and compatibility with functional additives such as phase-change materials and conductive fillers, although solvent toxicity and environmental impact remain challenges. Hybrid strategies that combine natural and synthetic polymers or employ benign solvent systems are increasingly important for producing durable, multifunctional, and skin-safe wearable textiles. Careful optimization of solvent choice, polymer concentration, and electrospinning parameters is therefore essential to balance fiber quality, functional performance, and sustainability, ultimately supporting the commercial translation of next-generation thermoregulating and sensor-enabled smart textiles.

Salts are commonly incorporated to increase solution conductivity, improve spinnability, and achieve finer and more uniform fiber morphologies. Reported examples include NaCl, LiCl, FeCl_3_, CuSO_4_, and AgNO_3_ [94]. Beyond their role in fiber formation, salts can impart additional functional properties relevant to wearable systems. For instance, metal-containing salts or nanoparticles can enhance electrical signal transmission in sensing layers [95], while AgNO_3_ provides both electrical conductivity and antimicrobial functionality [96]. From a thermoregulation perspective, such additives may also contribute indirectly by improving thermal conductivity or enabling integration with active heating or temperature-sensing elements.

Surfactants play a particularly important role in tailoring fiber morphology and surface properties. By reducing surface tension, surfactants such as Triton X-100 improve spinnability and fiber uniformity, while also facilitating the dispersion of functional additives including carbon nanotubes, graphene, or other nanomaterials [97,98]. Certain surfactants, such as sodium dodecyl sulfate, can increase solution conductivity [99], whereas others enable precise control over wettability [100]. This tunability is especially valuable for thermoregulating textiles, where hydrophilic surfaces promote sweat absorption and evaporation for cooling, while hydrophobic layers act as moisture or thermal barriers to reduce heat loss in colder environments [100]. Such functionality is essential for multilayer textile systems designed for adaptive thermal regulation.

Electrospun nanofibers can be functionalized using three primary strategies: polymer blending, incorporation of functional additives, or encapsulation, each offering distinct advantages for wearable smart textiles. Polymer blending enables synergistic effects that enhance thermal, mechanical, and sensing performance beyond that of individual components [101]. Incorporation of functional additives, such as conductive polymers, metal nanoparticles, carbon-based nanomaterials, or inorganic MXenes (listed in Table 3), can transform passive biomaterials into multifunctional systems, improving electrical conductivity, thermal management, and sensing capabilities. Conductive polymers such as PEDOT: PSS and polypyrrole provide lightweight flexibility but may degrade under repeated bending or washing and often require toxic solvents. Carbon-based nanomaterials, including graphene and carbon nanotubes, offer superior conductivity, thermal performance, and mechanical reinforcement; however, dispersion, aggregation, and potential cytotoxicity remain challenges. Metal nanoparticles such as silver and gold contribute high conductivity, antibacterial properties, and photothermal effects, though cost, environmental impact, and long-term stability must be considered. Inorganic MXenes deliver multifunctional conductivity and electrochemical responsiveness, but are prone to oxidation and require careful processing for durability. Encapsulation, typically achieved via coaxial electrospinning, allows incorporation of phase-change materials (PCMs) to absorb, store, and release thermal energy, providing passive thermal buffering while preserving a soft and breathable interface. Overall, while these strategies enable advanced thermoregulating and sensing functions, their integration demands careful optimization of biocompatibility, durability, washability, and environmental sustainability, highlighting the trade-offs between performance, safety, and practical applicability in next-generation wearable textiles.

Furthermore, the incorporation of thermally conductive fillers, such as carbon-based nanomaterials or MXenes, enables controlled heat distribution within electrospun mats. This capability is beneficial for both passive heat dissipation and integration with active thermal management systems, including electrically driven heating or temperature feedback mechanisms.

Once fabricated, electrospun nanofibers must be assembled into functional configurations suitable for wearable and thermoregulating textile interfaces. Common assembly strategies include layer-by-layer stacking and direct deposition onto functional substrates. Layer-by-layer stacking allows the construction of multilayer architectures with spatially separated functions, such as an inner hydrophilic cooling layer, a middle sensing or conductive layer, and an outer protective or insulating layer [98]. Direct deposition onto textile fabrics or polymer substrates ensures good adhesion, electrical contact, and preservation of stretchability—key requirements for wearable applications [79]. These hierarchical structures closely mimic natural skin thermoregulation mechanisms and enhance both user comfort and device functionality.

Finally, post-processing steps such as thermal annealing, chemical cross-linking, or encapsulation are often employed to improve mechanical durability, wash resistance, and long-term thermal stability, ensuring reliable performance of electrospun thermoregulating textile interfaces under real-world conditions.

## 4. Biocompatible Polymers with Thermoregulatory Potential

### 4.1. Natural Biopolymers for Passive and Active Thermoregulation

Electrospun materials from natural and synthetic polymers can be engineered for both passive (e.g., insulation, conduction) and active (adaptive heat regulation) thermal management, making them highly versatile for applications in electronics, textiles, and energy systems.

Radiant cooling textiles are becoming a practical and energy-efficient solution for passive personal thermal management, helping people stay comfortable outdoors. Passive systems rely on the properties of the electrospun fibers to control heat flow without external input. Electrospun mats of natural polymers (e.g., cellulose, silk fibroin, chitosan) provide low thermal conductivity due to their porous nanofiber structure, making them effective thermal insulators [18,107,108].

Active thermal management systems involve materials that respond dynamically to temperature changes. Electrospun fibers can encapsulate phase change materials (PCMs, such as paraffin or fatty acids) that absorb heat when temperatures rise and release it when they drop, enabling adaptive regulation. Active thermoregulation typically requires functional additives (e.g., PCMs, carbon nanotubes, graphene, metallic nanoparticles) [109,110].

Materials such as PCMs and thermally conductive films, although increasingly explored within the field, still exhibit inherently limited thermal transport properties. Consequently, the efficiency of heat transfer between the human body—the primary thermal source—and the cooling system remains constrained. Furthermore, most materials employed for personal thermal management are not derived from bio-based feedstocks. Even in cases where wood or cotton is incorporated as the structural matrix, their biocompatibility and prospects for large-scale commercialization have not been comprehensively assessed.

Natural biopolymer PCMs include lipid, lignin, polysaccharides, proteins, and other biopolymers. The advantages of natural polymers include eco-friendliness, biodegradability, and low toxicity, and they can also withstand temperature fluctuations, making them useful for passive thermal management [111].

Another important feature is that, when using biopolymers at high temperatures, crosslinking, the incorporation of various nanofillers, or blending different polymers can be employed to stabilize thermal performance.

#### 4.1.1. Silk Fibroin: High Thermal Conductivity, Breathable and Its Mechanical Properties

Compared with synthetic fibers, silkworm silk is naturally degradable, and products derived from it are environmentally friendly [112]. Natural silk fibroin (SF) exhibits tensile strength of 300–740 MPa and can absorb energy before tearing, giving it high strength [111,113]. SF is thermally stable (above 250°) [114], exhibits excellent biocompatibility and good biodegradability, and its degraded products are non-toxic [115,116,117]. Additionally, SF is easily processed to tune mechanical and structural properties and can be chemically functionalized (e.g., cross-linked or modified to impart new properties) [118,119].

SF has been widely used in tissue engineering [117], wound dressings. Composite biomaterials incorporating SF are designed to improve mechanical properties, particularly in humid environments. Emerging applications include 2D silk film electronics [115,116,117,118,119,120].

One study explored biologically derived silk fibroin films for the production of thermoregulatory patches. Experimental results demonstrated temperature reductions of 2.5 and 8.2 °C on simulated skin surfaces under outdoor and indoor conditions, respectively [116].

SF has also been utilized in sensors due to its biocompatibility, biodegradability, and low manufacturing costs. However, silk fibroin-based sensors alone have limited mechanical strength, electrical conductivity, or moisture resistance. Combining SF with aramid fibers forms a composite material that enhances sensor performance [117].

The thermal conductivity of silk fibroin fibers has also been investigated. A rarely studied axial-direction analysis revealed that thermal conductivity decreases as the temperature increases from 13 °C to 26 °C. At room temperature, SF exhibits higher thermal conductivity than most textile fibers [118].

#### 4.1.2. Cellulose and Its Derivatives: Porous, Hydrophilic, Moisture-Regulating

Cellulose, a natural biopolymer, contains many hydroxyls (-OH) groups, which make it inherently hydrophilic. This hydrophilicity gives cellulose excellent moisture absorption, swelling and wetting properties. These properties are advantageous for many applications, including hydrogels, sorbents, biomedical devices, but can be disadvantageous when water resistance, dimensional stability, or durability in humid conditions are required.

Cellulose chains form strong hydrogen bonds, giving the polymer a robust structure. A characteristic feature of cellulose is its semi-crystalline structure, consisting of a mixture of crystalline and amorphous regions. Additionally, cellulose exhibits excellent mechanical (elastic) properties under pressure, which are direction-dependent and influenced by crystal size. Increasing the number of hydrogen bonds significantly enhances mechanical strength and affects porosity [121,122,123,124,125].

Cellulose is biodegradable and non-toxic, making it suitable for applications in tissue engineering and regenerative medicine. Cellulose-based materials are commonly used in drug delivery systems, controlled/sustained release systems, excipients, hydrogels, scaffolds and for biomedical surfaces [123].

Cellulose-based antimicrobial coatings and films are used in textiles and packaging, while water-resistant cellulose materials find applications in medical devices, packaging, and diagnostic tools [126,127,128,129].

Most research has focused on balancing cellulose hydrophilicity. Scientists have developed cellulose hydrogels using ionic liquids or NaOH/urea solutions. The production of such hydrogels allows precise control over crystallinity, porosity and hydrophilicity, which is critical for the fabrication of sensors [119].

Other researchers have explored the addition of plasticizers, such as glycerol. Incorporating glycerol into regenerated films enhances flexibility, reduces stiffness, and significantly modifies water interactions, resulting in a disruption of the hydrogen bond network [130].

#### 4.1.3. Chitosan and Alginate: Antibacterial, Humidity Buffering, Compatible with Phase-Change Systems

**Figure 7:**
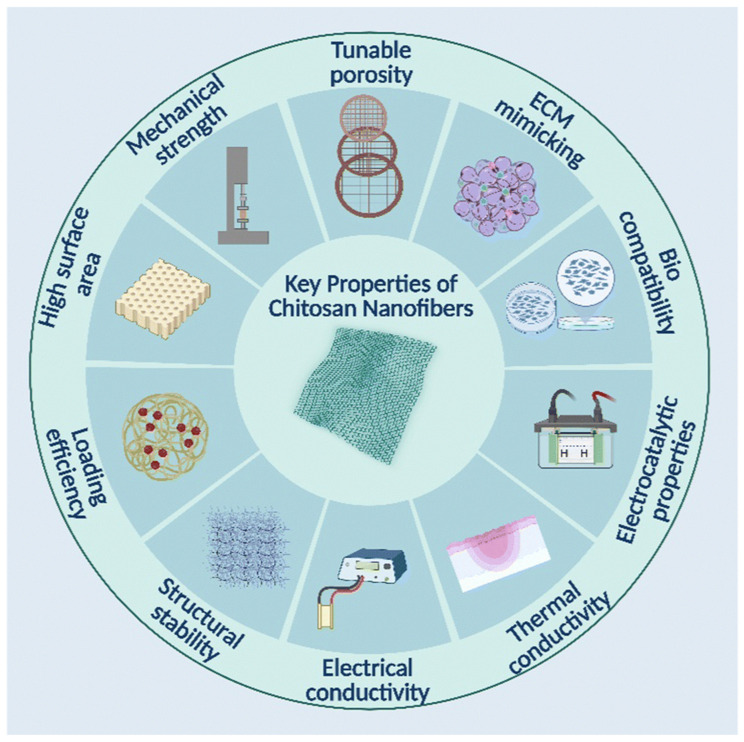
Key properties of electrospun chitosan nanofibers (reprinted and adapted with permission under a Creative Commons Attribution 3.0 Licence from Ref. [131]).

A significant role in chitosan’s antibacterial activity is played by its physicochemical properties, including cationic structure, molecular weight, degree of deacetylation, and concentration. Chitosan is easily processed: high molecular weight chitosan is less soluble, while low molecular weight chitosan is more soluble and often more bioactive and antimicrobial. Biological interactions with other substances depend on the degree of deacetylation. Viscosity is influenced by molecular weight and can be adjusted through temperature and concentration. Chitosan is biodegradable and can be broken down by lysozyme. It also exhibits excellent emulsification and water binding properties. These functional characteristics vary depending on its physicochemical profile. Due to the presence of reactive chemical groups, chitosan can be easily functionalized or chemically modified [130,131]. Key properties of electrospun chitosan nanofibers are presented in Figure 7.

Because chitosan contains a large number of amino and carboxyl groups, it can form chelate complexes with metals. In particular, the antimicrobial activity of silver (Ag) ions against Gram-negative and Gram-positive bacteria is well established. Chitosan-silver complexes are used in medicine, for example, as part of protective coatings, patches, and orthopedic products, helping reduce the risk of postoperative infection [132].

The main mechanism of chitosan’s antibacterial activity depends on its molecular weight, degree of deacetylation, physicochemical properties (concentration, pH, contact time), structure, and reactive hydroxyl groups. Chitosan can even inhibit bacterial growth by interacting with bacterial surface structures and forming metal chelates [133].

Chitosan can be applied in biomedical, food, cosmetic, and pharmaceutical sectors, for example as bandages and tissue engineering scaffolds, or even as a carrier component for anticancer drugs. In agriculture, it serves as a plant protection agent and growth stimulant, and it is also used in wastewater treatment. In the packaging sector, chitosan is applied for the production of biodegradable packaging [134,135,136,137].

Alginate is a high-molecular-weight biopolymer capable of forming gels with multivalent cations (e.g., Ca^2+^). Its viscosity is strongly dependent on molecular weight, pH, concentration, and composition and dissolves. Alginate dissolves well in water but is insoluble (or poorly soluble) in most organic solvents. A notable property is its mucoadhesiveness, allowing it to adhere to mucosal tissues. It can absorb large amounts of water. Due to its excellent biocompatibility, alginate is widely used in drug delivery, wound healing, and tissue engineering. In the food industry, it functions as a thickener, stabilizer, and gelling agent. Additionally, alginate is applied for cell encapsulation, immobilization, and microgranules, wastewater treatment (metal binding), biological recycling, and hydrogel production [138,139,140,141].

### 4.2. Synthetic Biopolymers with Enhanced Mechanical and Thermal Properties

#### 4.2.1. Polycaprolactone (PCL): Flexible Matrix, Blends Well with PCMs or Fillers

**Figure 8:**
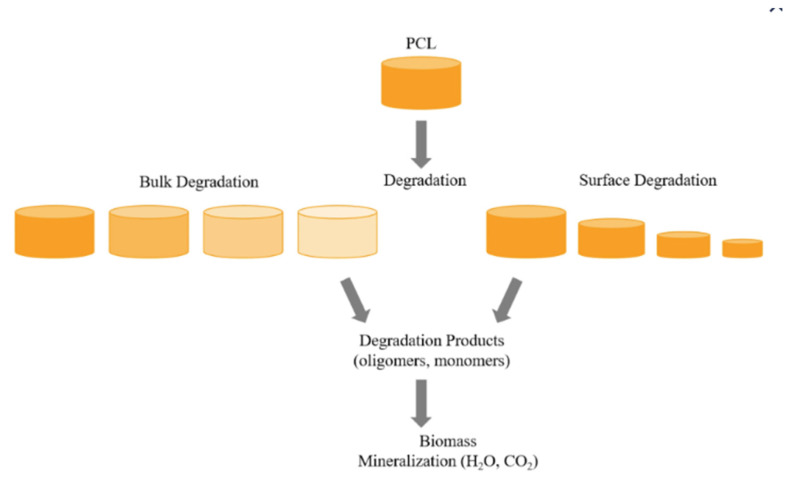
Bulk and surface degradation of PCL (reprinted and adapted with permission under a Creative Commons license (CC BY 4.0) from Ref. [144]).

The synthetic biopolymer polycaprolactone (PCL) is insoluble in water, but soluble in most organic solvents. It is very flexible, although its mechanical properties depend strongly on molecular weight and crystallinity. PCL is easily shaped and highly compatible with other polymers. It exhibits shape memory behavior due to its flexible chains and low melting point. PCL is biocompatible and its biodegradability is slow, taking up to about 3 years, and depends heavily on its intrinsic parameters. PCL undergoes degradation through two common pathways: (i) enzymatic degradation (also called surface erosion mechanism) and (ii) hydrolytic degradation (also known as bulk erosion mechanism). Bulk and surface degradation of PCL are presented in Figure 8. Surface enzymatic erosion of PCL causes substantial mass loss without significantly altering molecular weight (represents decrease of orange colour column size) because hydrolysis at the surface occurs faster than water can diffuse into the polymer bulk, resulting in gradual thinning from the outside inward (represents intensiveness of orange colour in the Figure 8. PCL’s hydrophobicity is relatively high [142,143,144,145].

This polymer is widely used in the biomedical field, including tissue engineering scaffolds, drug delivery systems, fand long-term implants (e.g., bone scaffolds). It is also applied in water purification and other industrial areas [143,144,145].

#### 4.2.2. Polyurethane (PU): Elastic, Breathable, Comfortable for Skin-Contact Sensors

The synthetic polymer polyurethane (PU) exhibits good elongation and tensile strength, excellent abrasion resistance, high elasticity and tear resistance. It also possesses very good thermal properties, works over a wide temperature range, good thermal properties, functioning over a wide temperature range, provides effective thermal insulation, good moisture resistance, and demonstrates excellent resistance to mechanical stress, fatigue, abrasion. However, this polymer is poorly degradable and is particularly susceptible to UV radiation and thermal degradation. Biocompatibility is limited, with only certain Pus being suitable for biomedical application PU offers versatile processing options in various forms, including spraying, foaming, molding. Recycling is limited to thermoplastic PUs, and its use in medical applications is generally restricted [146,147,148,149].

#### 4.2.3. Polylactic Acid (PLA): Thermally Insulating, Biodegradable, Forms Stable Nanofibers

Polylactic acid (PLA) is an insulating polymer that is highly biodegradable and can be composted under appropriate humidity and temperature conditions. Its thermal resistance depends on crystallinity and its mechanical properties are sensitive to processing conditions. PLA is compatible with a variety of fabrication methods, including sewing threads, 3D printing, thermoforming and foam molding, and finds applications in biomedical devices [150,151,152]. Similarly, PCL, due to its biobased and biodegradable nature, is an attractive alternative to traditional fossil-based insulators for specific applications. However, PCL often requires additional processing, such as annealing or incorporation into composites, to achieve enhanced thermal insulation performance [153].

### 4.3. Material Comparison and Performance in Thermoregulating Textiles

#### 4.3.1. Biocompatibility vs. Thermal Control Trade-Offs

**Figure 9:**
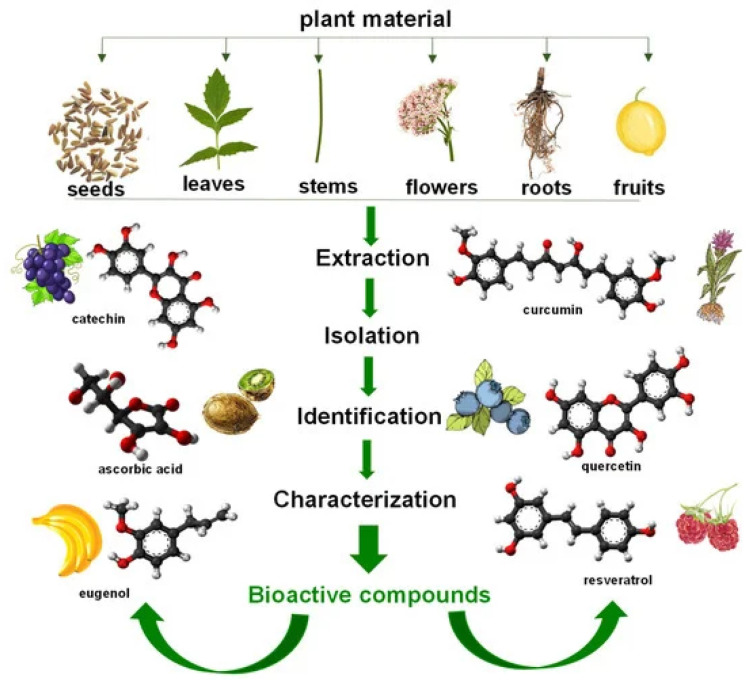
Schematic representation of general steps for extraction of bioactive compounds from plant materials (reprinted and adapted with permission under a Creative Commons license (CC BY 4.0) from Ref. [154]).

Biomaterials can be broadly classified into three principal categories based on their source and production pathway. The first category comprises polymers directly extracted or fractionated from natural biomass, including starch, cellulose, arabinoxylan, and lignin. The second category consists of polymers chemically synthesized from bio-derived monomers, such as polylactic acid (PLA) and cellulose acetate (CA). The third category encompasses microbially biosynthesized polymers, notably polyhydroxyalkanoates (PHAs) and various polysaccharides. The schematic representation of general steps for extraction of bioactive compounds from plant materials is presented in Figure 9 [154].

Biopolymers such as poly(caprolactone) (PCL), poly(ethylene glycol) (PEG), poly(lactic acid) (PLA), poly(lactic-co-glycolic acid) (PLGA), chitosan, and gelatin are commonly employed in electrospinning due to their high biocompatibility and ability to undergo biodegradation in physiological environments. However, these materials exhibit markedly different mechanical properties, which can influence the performance and structural stability of the resulting electrospun fibers [155]. PLA provides strength and rigidity for structural applications, while PEG is soft and flexible, supporting cell interactions but lacking mechanical support. PLGA combines strength and flexibility, making it versatile, and gelatin offers cell compatibility though with less strength than synthetics. Chitosan has low modulus but can be reinforced through cross-linking, and PCL is elastic and durable, enabling long-lasting fibers. Together, these materials allow electrospun fibers to be tailored for specific functional needs across diverse applications [155].

Advantages and limitations of natural and synthetic electrospun polymers are presented in Table 4.

Natural polymers provide comfort and eco-friendliness, while synthetics enable engineered thermal regulation. The future lies in hybrid electrospun textiles that merge both strengths for sustainable, high-performance thermoregulating fabrics.

#### 4.3.2. Blending Strategies (e.g., Natural/Synthetic Hybrids) for Combined Benefits

Blending natural and synthetic polymers into hybrid systems combines the eco-friendliness and biocompatibility of natural materials with the mechanical durability and tunable properties of synthetic polymers. Common strategies for blending include physical mixing, nanocomposite formation and coaxial electrospinning. These hybrid systems offer enhanced mechanical strength, improved thermal conductivity, better phase change stability, and controlled degradation, making them highly suitable for thermal energy storage, thermoregulating textiles, and other advanced functional materials.

A notable example is coaxial electrospinning, which produces core–shell nanofibers: natural polymers form the shell to ensure biocompatibility, while synthetic polymers or PCMs in the core provide thermal regulation [158,159,160].

These hybrids can be fabricated into lightweight, breathable structures suitable for smart textiles, wearable devices and biomedical applications. Their improved heat storage and release capabilities support personal thermal regulation and thermal energy storage systems. By combining natural polymers for reduced environmental impact with synthetics for robustness, hybrid systems create balanced, high-performance materials optimized for long-term use [161,162,163,164].

## 5. Thermoregulation Strategies Using Electrospun Biomaterials

### 5.1. Passive Thermoregulation via Structural Design

Electrospinning is a widely used and cost-effective method for fabricating membranes with tailored structures for passive thermoregulation through precise control fiber morphology and porosity. The high surface area, tunable porosity, and controllable morphology of electrospun nanofibers make them particularly well-suited for personal thermal management applications [165,166].

**Figure 10:**
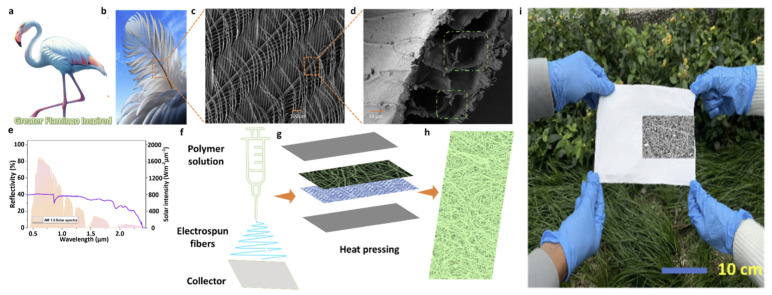
Inspiration, morphology, photographs, and schematic detailing the fabrication of PAC@T, a hierarchical polyacrylonitrile–alumina–cellulosic cotton knit fabric (PAN–Al2O3–CKF) radiative cooling smart textile. (**a**) Schematic of a greater flamingo (*Phoenicopterus roseus*). (**b**) Feather of *Phoenicopterus roseus*. (**c**,**d**) Morphological representation of *Phoenicopterus roseus* feathers showing barbs and barbules. (**e**) Solar reflectance spectra of *Phoenicopterus roseus* feather. (**f**–**h**) Typical electrospinning setup and heat pressing approach used for PAC@T fabrication. (**i**) Photograph of electrospun nanofabric Reprinted with permission from Ref. [167]. Copyright © 2025 ACS Publications.

Nature-inspired designs have emerged as a powerful strategy for creating effective thermoregulating textiles. For example, the feather structure of *P. roseus* has been mimicked to produce a PAC@T smart textile that has cooling properties which are achieved due to its micro and nano fibers and pores (see Figure 10). This type of material exhibits breathability, durability, and enhances mechanical strength [166]. Another nature inspired textile that can be used for personal thermal management as well as for protection against fire was inspired by hyper-white beetle scales. This material has flame retardants with high whiteness and pore structures inspired by biomimetic structures that are introduced into polyurethane coatings. Composite textiles are prepared by a scraping-coating technique [167].

**Figure 11:**
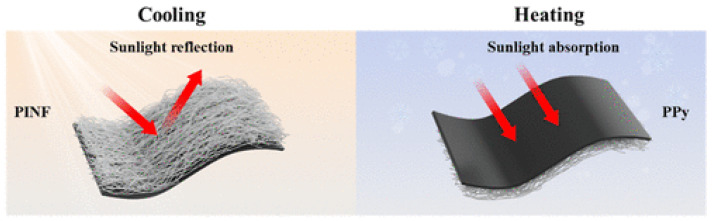
Hierarchical nanofibrous structure enabling dual-mode thermal behavior. Reprinted with permission from Ref. [169]. Copyright © 2025 ACS Publications.

Another thermoregulating textile was analyzed by Q. Gao with colleagues—a smart dual-sided nonwoven textile with coating of PI nanofibrous membranes with AgNWs. Results of tests showed that this material has electrical and thermal properties suitable for smart textiles with personal thermal management. Compared to other AgNW coated textiles it displayed better IR reflection performance. Increasing the AgNW content was observed to promote the formation of highly interconnected AgNW networks, characterized by exceptionally low sheet resistance (0.23 Ω sq^−1^) and strong infrared reflectance exceeding 80%, substantially outperforming conventional textile materials. The dual-layer nonwoven structure, when oriented with the AgNW-coated surface outward, exhibits a favorable passive thermoregulatory effect [168]. Also, dual-mode membrane- porous composite (see Figure 11) aimed at passive thermoregulation created by Q. Zhang with colleagues can be used for thermal isolation. Polyimide nanofiber membrane, made by electrospinning, with incorporated fluorine-containing and aliphatic structures shows a possibility for materials to adapt to seasonal and weather changes meaning it has good thermal isolation and can transfer heat when needed [169]. The polyimide composite membrane exhibits a hierarchical nanofibrous structure, combining micro and nanos pores that reduce solid conduction and suppress internal air convection. Its chemical composition, including fluorine containing and aliphatic segments, enables dual-mode thermal behavior, allowing the fabric to either retain heat or enhance radiative cooling depending on environmental conditions [169,170].

In another article, a composite that is produced through electrospun polymer matrix combining the fibrous polymer matrix with SiO_2_ aerogel was investigated. The material has strawberry inspired structures for better thermal insulation and absorption properties. It was discovered that integrated SiO_2_ aerogel helps to achieve high porosity which enhances thermal insulation by trapping air within the structure. The strawberry like composite membrane demonstrates a low thermal conductivity of 0.028 W/m·K, indicating excellent thermal insulation performance [171]. Silica/polyimide composite nanofiber membranes via an electrospinning process can enhance the thermal insulation performance of conventional polyimide nanofiber membranes. According to T. Zhuo by doping with SiO_2_ nanoparticles, a low thermal conductivity of the membrane is achieved. Thermal resistance properties appear, because of the resistance to heat transfer between the SiO_2_ NPs and PI nanofibers. This result indicates that the membrane can prevent the heat of fire from damaging materials superhydrophobicity [172]. Also, it was found out that when PAMPS nanofibers are added to PU, it increases the number of pores and decreases pore diameter. This method helps to create windproof (thermal insulation) material, because of the decreased pore diameter. Also, it was stated that for increasing thermal insulation, it is better to use sequential electrospinning mode to produce hybrid layers (structure containing different materials to enhance properties) than simultaneous electrospinning mode [173].

### 5.2. Moisture-Driven Thermal Management

Wearable devices as well as smart textiles pose additional requirements of thermal comfort and safety. It may limit body heat dissipation and may lead to thermal stress and discomfort even at moderate exposure temperatures. Effective regulation of perspiration is essential, as the evaporation of sweat requires heat absorption to stabilize body temperature [174,175,176]. Decreased or increased localized sweat loss is indicative of hyperhidrosis or hypohidrosis and often assists in stroke diagnosis [177]. An increasing interest in environmentally friendly materials has motivated industrialists to develop and use biopolymers for various applications such as humidity buffering.

**Figure 12:**
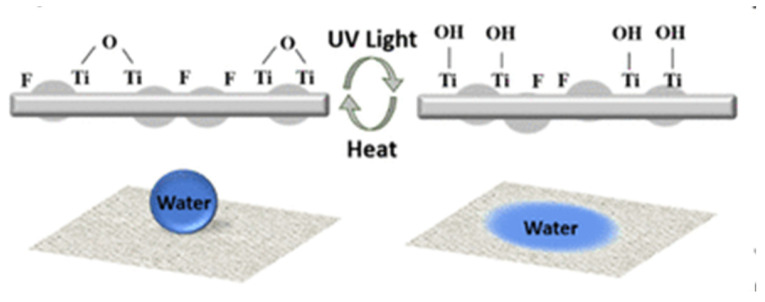
The mechanism of the conversion process of wettability of PVDF + TiO_2_ fibers (reprinted and adapted with permission under a Creative Commons license (CC BY 4.0) from Ref. [179]).

Y. Zhang with colleagues created a superhydrophobic self-cleaning PTFE nanofiber membrane. The material SNM-PTFE was created through one-step electrospinning process and achieved superhydrophobic properties by stabilizing the SiO_2_ aerogel protrusions. This material exhibits exceptional chemical stability, remarkable resistance to elevated temperatures, pronounced water repellency, and highly effective self-cleaning behavior, all of which arise from its engineered surface architecture composed of micro- and nanostructured features [178]. G. Parisi and colleagues in [179] examined electrospun polyvinylidene fluoride (PVDF) fiber meshes incorporating a photoresponsive, switchable surface capable of transitioning from a hydrophobic to a hydrophilic state upon UV irradiation, and subsequently reverting to hydrophobicity following thermal treatment (see Figure 12). These properties help collect and release humidity from the material when needed, because of different atmospheric conditions [179].

Hydrophilic methylcellulose–polyvinyl alcohol biopolymer formed from sugarcane exhibits excellent humidity buffering behavior, with high water absorption, strong moisture retention, and low vapor transmission. These properties result from the abundant hydroxyl groups in methylcellulose, PVA, and starch, which form hydrogen bonds with water. These kinds of materials are a possibility for industrial application of sugarcane base to form various products [180]. Also, super hydroscopic and fast moisture absorption can be achieved by using facile and two-step electrospinning. The moisture absorption properties are achieved by coating nanofiber with LiCl via impregnation. Structure plays a crucial part in determination of the properties of fiber. The porous nanofibrous structure significantly increases the moisture absorption and transport rates [181].

For enhancing thermal comfort sweat management innovations such as moisture pumping textiles can be used. Such materials help to travel moisture from the skin to the environment. This can be realized by arranging hydrophobic polyester coils and hydrophilic microfiber polyester coils on a bi-layer knitted fabric (moisture travels through the hydrophobic layer into the hydrophilic layer). Using microfiber polyester helps to achieve better transition speed for this type of material, so that transmission speed would match the diffusion speed [182]. Another way that moisture pumping can be achieved in fabrics is by using Janus (membrane that has opposite properties on each side membranes [183]). X. He with colleagues analyzed a material—electrospun polyurethane nanofiber onto superhydrophilic gauze. As the material mentioned before, it has a moisture- pumping mechanism, ensures wearers comfort and is a suitable option for wearable health monitoring applications [184].

### 5.3. Thermoresponsive Electrospun Materials for Active Thermal Regulation

**Figure 13:**
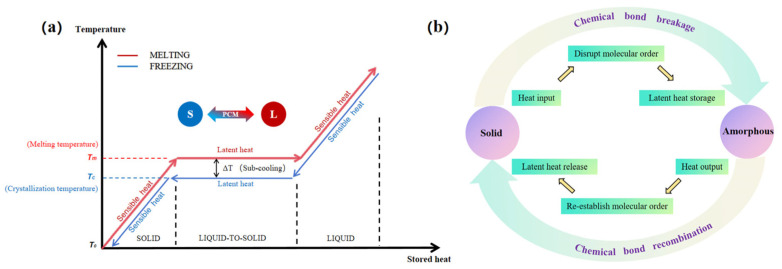
(**a**) Schematic diagram of the working of PCMs; (**b**) Phase transition process of SSPCMs (reprinted and adapted with permission under a Creative Commons license (CC BY 4.0) from Ref. [148]).

The use of phase change materials (PCMs) has been widely reported as an effective strategy to improve thermal comfort and reduce thermal stress in wearable applications [185]. PCMs function as heat reservoirs by absorbing and releasing thermal energy through a solid–liquid phase transition, which is associated with high latent heat storage capacity [148,186]. The schematic diagram of PCM work and phase transition of Polyurethane solid–solid phase change materials (SSPCMs) are presented in Figure 13. To enable their incorporation into textiles, PCMs are frequently microencapsulated (μPCMs), preventing leakage during phase transition and allowing their integration into fibrous systems [187]. Microencapsulated PCMs are particularly suitable for textile applications, as their polymeric shells preserve structural integrity under repeated thermal cycling and mechanical deformation.

Several fabrication approaches have been explored to incorporate μPCMs into fibres. Ahn et al. in [186] demonstrated the successful production of μPCM-polymer fibre composites using conventional dry-jet wet-quench spinning techniques, achieving PCM loadings of up to 80 wt %. While high μPCM content resulted in reduced mechanical strength and elasticity, the thermal energy storage performance of μPCM-cellulose acetate and cellulose fibres indicated strong potential for smart textile applications requiring passive thermoregulation. Industrial studies have further confirmed the durability of PCM-loaded fibres, reporting stable thermal performance over more than 100 heating-cooling cycles [188].

Despite their effectiveness, most commercially available PCMs are derived from non-renewable sources, raising environmental concerns. Consequently, increasing attention has been directed toward bio-based PCMs. Natural fatty acids such as myristic, palmitic, and stearic acids, as well as octadecanol, have been encapsulated using gelatin–pectin biopolymer shells, producing sustainable PCM composites with excellent thermoregulation properties and no leakage during phase transition [189]. Similarly, palmitic acid encapsulated within biodegradable polylactic acid (PLA) shells, using poly(vinyl alcohol) (PVA) as an emulsifier, has demonstrated effective thermal regulation while improving environmental compatibility [190]. These developments highlight the growing shift toward sustainable and biocompatible PCM systems.

**Figure 14:**
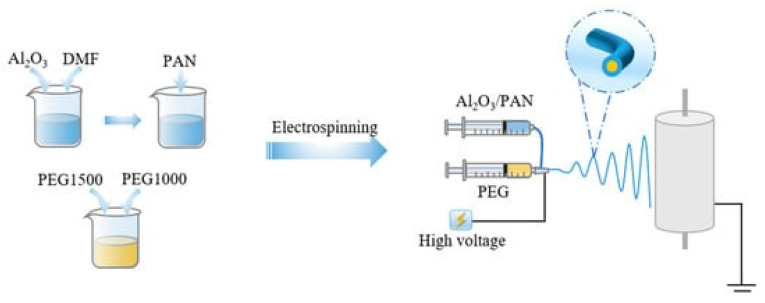
Schematic diagram of the preparation of phase change thermoregulated nanofiber membranes by coaxial electrospinning (reprinted and adapted with permission under a Creative Commons license (CC BY 4.0) from Ref. [191]).

Electrospinning has emerged as a particularly versatile platform for integrating PCMs into textiles due to its high porosity, conformability, and compatibility with sensitive materials. Huang et al. in [191] developed coaxial PAN/PEG electrospun fibres doped with Al_2_O_3_ nanoparticles (see Figure 14), achieving stable heat capacity and enhanced fibre durability.

Reviews by McCord et al. in [192] and Das et al. in [102] further demonstrated that core–shell electrospun designs significantly improve PCM encapsulation efficiency and thermal cycling stability, especially when biocompatible shells such as PVA or polyurethane are used. More recent work by Zhang et al. in [193] reported a hybrid electrospun membrane incorporating PVA, PCM microcapsules, and nano-silica, achieving both thermal energy storage and passive cooling through solar reflectance and mid-infrared emission. Crosslinking strategies, such as photo-crosslinking, have also been shown to enhance PCM retention and wash durability, maintaining thermal functionality after repeated laundering cycles [194].

Beyond passive thermal buffering, smart textiles increasingly incorporate materials that provide real-time thermal feedback. Thermochromic materials, which reversibly change colour in response to temperature variations, offer an intuitive and non-intrusive method for thermal sensing. Lee et al. in [195] developed thermochromic electric heating textiles in woven and knitted structures, demonstrating that double-layer woven fabrics exhibit superior heating performance, tensile strength, and clearer colour transitions compared to knitted counterparts. The effectiveness of colour change was found to depend not only on fabric structure but also on yarn composition and insulation, with soybean yarns showing particularly pronounced thermochromic responses.

**Figure 15:**
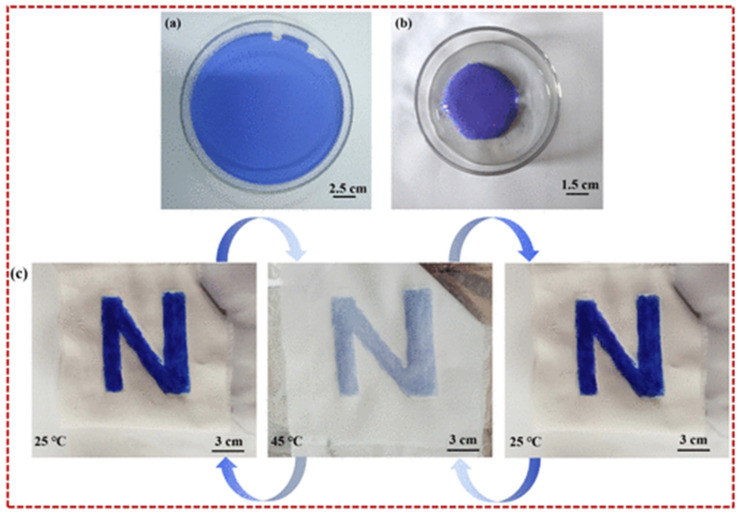
(**a**) Powder of RTPCMs-2, (**b**) coatings of RTPCMs-2, and (**c**) reversible discoloration of RTPCMs-2 on fabrics [201]. Copyright © 2024 ACS Publications.

Recent advances have extended thermochromic functionality into electrospun systems. Supian et al. in [196] highlighted the rapid development of reversible thermochromic polymer nanocomposites, while Ma et al. in [197] demonstrated electrospun membranes containing leuco dyes capable of colour change under skin-relevant temperatures. Simpler approaches using commercially available thermochromic powders embedded in PMMA nanofibres have also shown effective colour transitions [198]. However, challenges remain regarding wash-fastness and long-term durability. Solutions such as flexible binders, sol–gel coatings, and plasma surface treatments have been shown to improve dye fixation and resistance to water exposure [199,200]. Recent studies suggest that combining thermochromic materials with PCMs within composite fibre architectures can simultaneously provide visual feedback and thermal regulation (see Figure 15) [201,202].

Another emerging class of thermoresponsive materials is temperature-sensitive hydrogels. Hydrogels based on poly(N-isopropylacrylamide) (PNIPAM) exhibit a lower critical solution temperature (LCST) near 32 °C, enabling rapid volumetric contraction or swelling in response to small changes in skin temperature. Huang et al. in [203] developed a temperature-responsive self-contracting nanofibre/hydrogel composite using electrospun poly(lactic acid-co-trimethylene carbonate) (PLATMC) nanofibres combined with methacrylate gelatin hydrogel layers. Although hydrogels typically suffer from weak mechanical properties, biaxial orientation techniques have been shown to significantly enhance their mechanical strength and durability [204]. Recent studies further demonstrate that PNIPAM-based blends with chitosan, hyaluronic acid, or alginate improve biocompatibility, mechanical resilience, and self-recovery after deformation [205,206].

**Figure 16:**
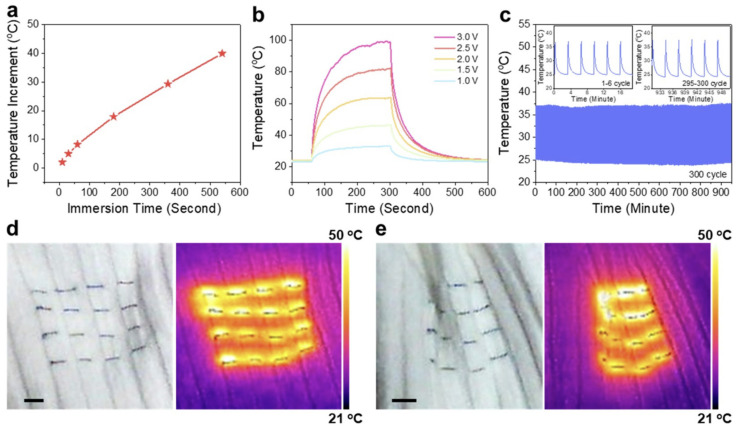
Conductive MXene@A fibers for wearable thermal management. (**a**) Increase in surface temperature of MXene@A fibers from a 1 wt % CaCl2 bath with different MXene immersion times under Joule heating at 2.0 V for 4 min. (**b**) Temperature–time profiles of MXene@A fiber (fabricated from 540 s MXene immersion) under Joule heating at different voltages. (**c**) Temperature–time profiles of MXene@A fiber (fabricated from 540 s MXene immersion) under repeated Joule heating at 2.0 V for 10 s over 300 cycles. Insets show the first and the last six cycles. (**d**,**e**) Optical photographs (left) and IR thermal images (right) of a sweater knitted with MXene@A fibers (1 wt % CaCl2 bath, 60 s MXene immersion) under Joule heating at 2.0 V at (**d**) relaxed and (**e**) bent state. Scale bar is 1 cm [105]. Copyright © 2021 ACS Publications.

In contrast to passive systems, active heating textiles enable on-demand thermal regulation through Joule heating. Nanoconductive fibres incorporating materials such as MXenes have demonstrated excellent electrical and photothermal heating performance while maintaining flexibility and durability under repeated mechanical deformation (see Figure 16) [105,106]. However, challenges related to oxidation stability and skin safety persist. Encapsulation strategies using hydrophobic biopolymers have been proposed to preserve conductivity while minimizing skin irritation [207]. Importantly, Joule heating systems must balance electrical performance with breathability and comfort, particularly when integrated with PCMs or hydrogels.

Despite significant progress, comparative studies evaluating these thermoresponsive systems under realistic use conditions, such as sweating, washing, bending, and long-term skin contact, remain limited. Long-term biocompatibility data are especially scarce, particularly for nanomaterial-based systems. As a result, current research is increasingly focused on multimodal textile architectures that integrate PCMs, thermochromic feedback, hydrogels, and conductive heating within layered or coaxial electrospun structures. These multifunctional designs offer a promising pathway toward adaptive, durable, and sustainable smart textiles capable of dynamic thermal regulation, user feedback, and enhanced wearer comfort.

## 6. Thermally Integrated Multifunctional Sensor Systems

Smart, wearable textiles are fabrics, with ability to sense external stimulation and to respond to it in a certain way. The external stimuli can be thermal, mechanical, chemical, electrical, magnetic, optical, etc. [208,209], with the integration of functional materials such as silver nanoparticles [210], graphene [103], or conductive polymers [211], nanofiber textiles can sense, respond, and interact with the user’s environment or body. This review part discusses about electrospun conductive biomaterial-based composites for temperature sensing. Electrospinning is a technique, which allows the incorporation of functional materials, such as nanoparticles, into the fibers, creating composites suitable for various applications, including thermal regulation.

**Figure 17:**
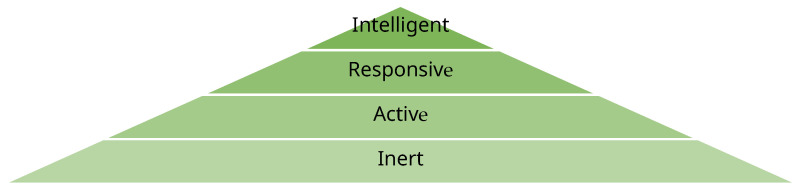
Levels of biomaterials smartness.

There are four levels of smartness for biomaterials, namely inert, active, responsive, and autonomous or intelligent (Figure 17).

Inert biomaterials provide biocompatibility without eliciting adverse effects, meaning they do not trigger toxic or harmful responses within the body. Active biomaterials enable a unidirectional, non-regulated release of therapeutic agents. Responsive biomaterials are capable of detecting specific environmental or physiological cues and subsequently initiating therapeutic release. Autonomous biomaterials not only sense such signals but also dynamically adjust their functional properties in response to changing conditions, thereby sustaining the delivery of enhanced or alternative therapeutic modalities [212,213].

In nature, biopolymer exists in the form of proteins, cellulose, starch, gelatin, chitosan (CS), polysaccharides, collagen, and nucleic acids Polymers by itself cannot improve thermal conductivity [214,215,216]. Thus, so many researchers are functionalizing bio composites by embedding various conductive additives.

### 6.1. Conductive Biomaterial-Based Composites for Temperature Sensing

Body temperature is a fundamental physiological indicator of human health. Although the body maintains a narrow thermal range under normal conditions, even slight deviations often signal the onset or progression of disease. Because temperature fluctuations accompany a wide variety of pathological states, continuous and accurate monitoring has become a central objective in the development of wearable biomedical devices. Numerous studies have demonstrated that wearable temperature sensors must combine high sensitivity, mechanical stability, and long-term reliability to function effectively in real world environments [93,217].

To meet these requirements, researchers have increasingly turned to advanced materials and fabrication strategies capable of producing flexible, skin conformal, and multifunctional sensing platforms. This chapter provides a comprehensive overview of the principles, materials, and emerging electrospun systems that underpin modern wearable temperature sensors. A sensor operates by converting a nonelectrical physical quantity into an electrical signal that can be processed, quantified, and interpreted. A broad range of materials—including semiconductors, ceramics, metals, and organic polymers—can serve as sensing elements. The intrinsic properties of these materials determine not only the sensitivity and stability of the device but also its potential to integrate multiple sensing functions within a single platform [218].

Tactile sensing, a closely related field, involves the spatial measurement of diverse stimuli such as pressure, strain, shear, temperature, and humidity [219]. Among these, temperature remains a central physiological parameter for real time monitoring of vital signs [220,221]. The convergence of tactile and thermal sensing in wearable systems has motivated the exploration of materials that are flexible, biocompatible, and capable of multimodal signal transduction.

**Figure 18:**
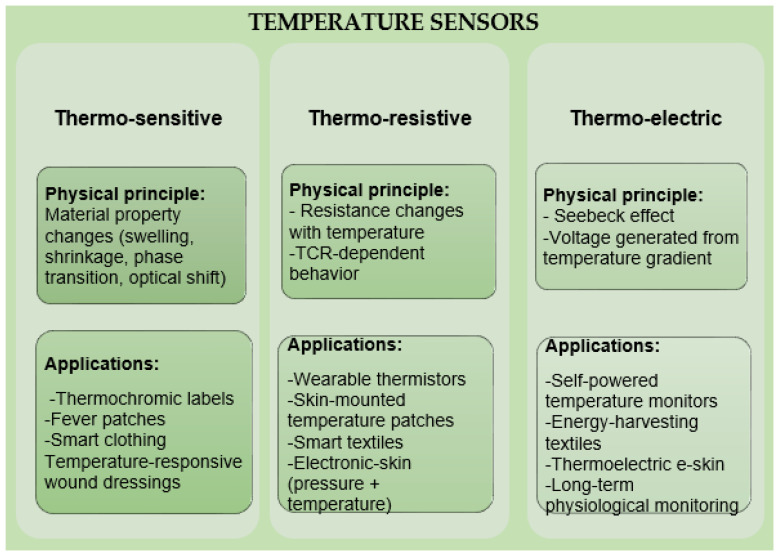
Temperature sensors with physical principles and applications.

Electrospinning has emerged as a powerful technique for producing nanofibrous materials with high surface area to volume ratios, tunable porosity, and excellent mechanical flexibility. When conductive nanomaterials—such as carbon nanotubes (CNTs) or graphene—are incorporated into biopolymer matrices, the resulting composites exhibit temperature dependent electrical behavior suitable for wearable sensing applications [222]. These electrospun conductive composites respond to thermal changes through variations in electrical conductivity, enabling lightweight, breathable, and skin compatible temperature sensors. Their structural versatility also allows integration into textiles, patches, and other conformal formats required for continuous physiological monitoring. Temperature sensors used in wearable systems typically rely on one of three primary mechanisms: thermo-sensitive mechanisms, thermo-resistive mechanisms and thermo-electric mechanisms (Figure 18). Thermo-sensitive sensors detect temperature through changes in the electrical resistance of the active layer. These resistance variations arise from temperature induced modifications in charge transport pathways within the sensing material [223,224,225,226].

Thermo resistive sensors operate on the principle that electrical resistance varies with temperature due to changes in material conductivity. The temperature coefficient of resistance (TCR) is a key parameter governing their performance. For example, Shin et al. [227] fabricated a NiO based thermoresistive sensor by coating NiO nanoparticle ink onto PET, demonstrating the importance of rapid response and long-term stability in wearable thermistors. Thermoelectric sensors exploit the Seebeck effect, in which a temperature gradient generates an electrical voltage. This mechanism enables self-powered sensing in some configurations, making thermoelectric devices attractive for long term wearable applications [227]. Nanocomposite systems offer enhanced performance due to synergistic interactions between polymer matrices and nanoscale fillers. Ben Shimon and Ya’akobovitz [104] developed flexible, biocompatible temperature sensors using carbon nanotube (CNT PDMS) composites. Thermal mismatch between CNTs and PDMS induces strain within the conductive network, altering electrical pathways and enabling temperature dependent resistance changes. These sensors exhibit excellent flexibility, low weight, and high reproducibility under repeated mechanical loading, making them suitable for on skin deployment. Material selection plays a critical role in sensor comfort and durability. Natural polymers (cellulose, silk, chitosan) provide biodegradability and skin friendliness. While, synthetic polymers (polyurethane, polyacrylonitrile) offer mechanical strength, elasticity, and long-term stability under harsh conditions [228,229]. Electrospinning and electrospraying are versatile techniques for functionalizing thermal textiles. Electrospun phase change fibers offer several advantages, including the elimination of encapsulation steps, controllable fiber dimensions, and cost-effective processing [230]. Besides CNT, graphene, boron nitride and silicon nitride nanoparticles, phase change materials are very popular components in thermal management systems [231]. PCMs are widely used in thermal management systems and can be classified: by chemical nature as organic PCMs (o PCMs), inorganic PCMs (io PCMs) and eutectic PCMs (eu PCMs). By physical behavior as solid–solid, solid–liquid, solid–gas and liquid–gas PCMs [192,232,233,234].

PCMs are particularly attractive for smart textiles due to their ability to regulate heat flow without significant temperature change. Biopolymer based PCMs have been incorporated into medical products such as bandages, where they absorb and store body or external heat and subsequently release it gradually [235]. Despite their advantages, PCMs face challenges such as leakage and fluidity during melting. Electrospinning provides an effective strategy to confine PCMs within form stable nanofibers, improving reliability and expanding applicability [192]. Incorporating conductive fillers such as CNTs or graphene further enhances thermal conductivity, enabling both passive heat dissipation and active temperature control [232]. A comprehensive overview of electrospun PCM reinforced nanofibers and their additives is presented in Table 5.

Wearable sensors can be organized into four major categories [218]: single multifunctional sensors, planar integrated sensors, three-dimensional assembled sensors, stacked or hybrid integrated sensors. For temperature sensing, carbon-based nanomaterials-including CNTs, graphene, boron nitride, silicon nitride nanoparticles, and carbon nanofibers-are frequently integrated into electrospun biopolymer matrices due to their exceptional electrical and thermal performance [246,247]. Stacked or hybrid architectures integrate multiple sensing modalities—such as temperature, pressure, and humidity—within a single layered device. These systems use resistive, thermoelectric, or other mechanisms to achieve simultaneous, compact, and interference resistant detection.

As an example of stacked or hybrid structure multifunctional temperature sensor Yu X et al. [219] demonstrated a multifunctional tactile sensor capable of detecting pressure, temperature, and material type concurrently, illustrating the potential of layered integration for real time health monitoring.

Electrospun conductive materials, particularly those incorporating CNTs or graphene, are highly promising for thermal management due to their high conductivity and structural tunability. When integrated into stacked or hybrid systems, they enable both passive heat dissipation and active temperature regulation, supporting complex sensing tasks in next generation wearable electronics [218].

Wearable temperature sensing technologies have advanced significantly through innovations in materials science, nanocomposites, and electrospinning. The integration of conductive nanomaterials, biopolymers, and phase change materials has enabled flexible, lightweight, and multifunctional sensing platforms capable of real time physiological monitoring.

### 6.2. Simultaneous Antibacterial, Breathable, and Thermal Properties

Electrospinning has emerged as a versatile, cost-effective, and efficient technique for producing micro- and nanofibrous structures with controlled morphology and composition [213,248,249,250]. Owing to their thin, lightweight nature, electrospun nanofiber mats can be seamlessly integrated into wearable systems, including textile fabrics. These nanofibrous platforms are particularly suitable for sensor applications, enabling the detection of physical parameters such as movement, temperature, and humidity [73]. This foundational capability makes electrospinning an ideal method for engineering multifunctional materials that combine thermal regulation, breathability, and antibacterial performance. Biopolymers enriched with functional additives offer a promising route toward materials that simultaneously exhibit thermoregulating and antibacterial properties. Such multifunctionality is typically achieved by incorporating phase change materials (PCMs) to manage heat flow and antimicrobial agents-such as metal nanoparticles or bioactive compounds-to inhibit bacterial growth. This dual-function design enables the development of advanced smart textiles suitable for medical, environmental, and wearable applications. Several studies have demonstrated the effectiveness of integrating PCMs and conductive or reinforcing additives into electrospun fibers. Wu et al. [244] fabricated PEG/PVA composite membranes via green electrospinning, achieving excellent flexibility, breathability, and thermal regulation. The incorporation of carbon nanotubes (CNTs) significantly enhanced mechanical strength and increased thermal conductivity by 40.4% at only 1.5 wt % CNT loading. The membranes exhibited a practical phase-change temperature range (26.9–38.9 °C) and high latent heat values, confirming their suitability for wearable thermal management.

**Figure 19:**
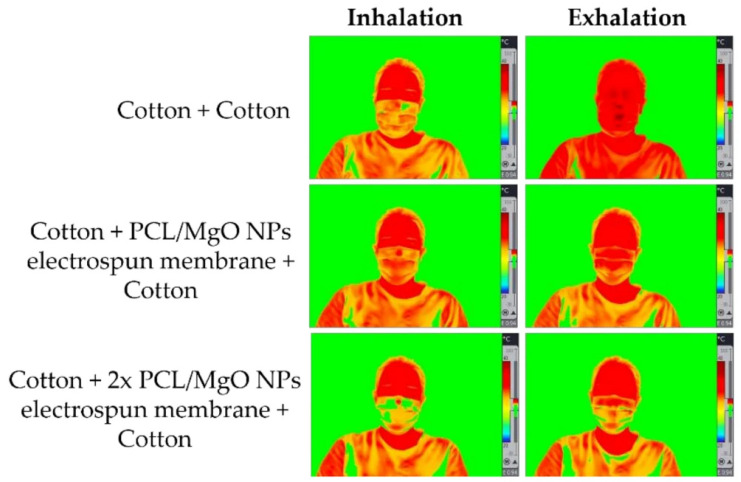
An example of facial temperature distribution for each condition (reprinted and adapted with permission under a Creative Commons license (CC BY 4.0) from Ref. [256]).

Similarly, Qin et al. [158] developed antibacterial, thermoregulating textiles using coaxial electrospinning, with PAN/curcumin forming the sheath and n-octadecane serving as the PCM core. These materials demonstrated strong potential for applications in clothing, food preservation, and biomedical products. Wang et al. [159] further advanced this field by synthesizing curcumin-based polyurethane (Cur-PU) films with excellent antibacterial activity and systematically characterized their thermal, mechanical, and biocompatibility properties. Curcumin has been widely explored as a natural antimicrobial agent in electrospun systems. Leng et al. in [251] encapsulated curcumin in PCEC nanoparticles and incorporated them into PVA/collagen composite films, demonstrating strong antibacterial performance. Additional studies have produced curcumin-loaded PLA/PVP nanofibers [252] and chitosan–collagen nanofibrous mats [253], both showing significant potential for wound healing and biomedical applications. Lin et al. in [254] fabricated hydrophilic HCP and HCPG nanofiber membranes that effectively reduced bacterial adhesion, particularly against *S. aureus*. Likewise, Khanzada et al. in [255] developed Aloe Vera/PVA nanofibers with inherent antibacterial activity, highlighting the versatility of natural bioactive compounds in electrospun systems. Nanoparticles have also been employed to impart multifunctionality to electrospun membranes. Costa et al. in [256] produced biodegradable PCL membranes functionalized with Ag, TiO_2_, and MgO nanoparticles, achieving high filtration efficiency and strong antibacterial effects. Notably, integrating PCL/MgO membranes within cotton layers improved thermal comfort, demonstrating the synergistic benefits of nanoparticle incorporation (see Figure 19).

Peng et al. in [27] extended these concepts to electronic skin (e-skin) applications by developing a flexible, breathable, biodegradable nanofiber-based triboelectric system incorporating Ag nanowires. This work illustrates the potential of multifunctional nanofibers in next-generation wearable electronics. Further innovations have focused on enhancing UV protection, waterproofing, and breathability alongside thermal regulation. Wang et al. [257] created anti-UV, thermo-regulating membranes using ZnO nanoparticles in coaxially electrospun octadecane/PAN fibers. Additional work demonstrated strong thermal energy storage in PEG/PA6/TiO_2_ composites [247,257]. Yi et al. [258] produced waterproof-breathable CNT-loaded membranes that blocked liquid water while allowing sweat vapor to escape, improving wearer comfort.

**Figure 20:**
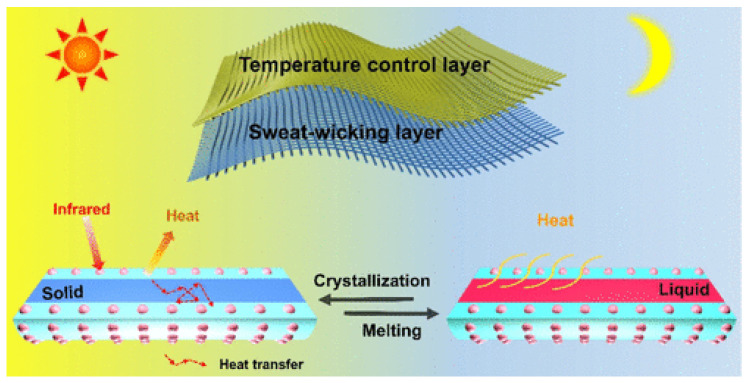
Mechanism diagram of smart textiles with the PVDF core–shell structure. [259]. Copyright © 2021 ACS Publications.

**Figure 21:**
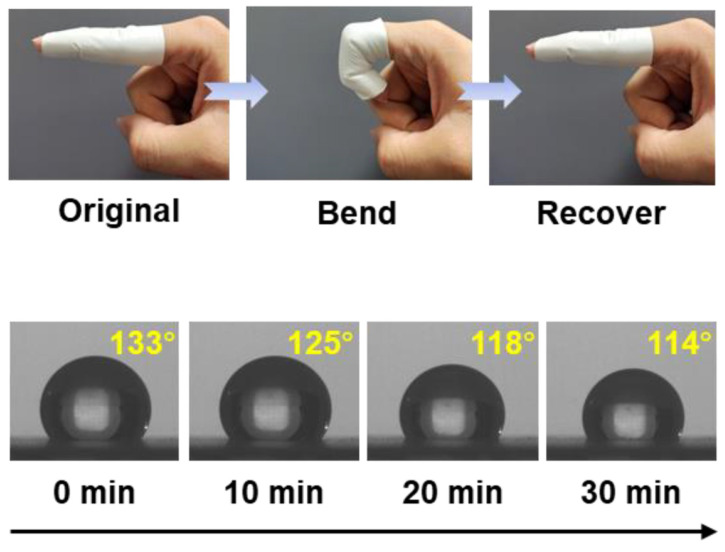
The elasticity and dynamic measurements of water droplet permeation of the nanofibrous membranes prepared from 20 wt % PBSe/PO3G-BPU solution (reprinted and adapted with permission under a Creative Commons license (CC BY 4.0) from Ref. [260]).

Xu et al. in [259] developed dual-mode fabrics combining n-eicosane/PVDF/Cu_7_S_4_ membranes with electrospun PAN layers, achieving both thermal management and breathability (see Figure 20). Feng et al. in [160] fabricated coaxial PU/PEG membranes with temperature-responsive moisture permeability, enabling sweat evaporation and thermal buffering. Zhang et al. in [260] produced PBSe/PO3G-BPU membranes with excellent waterproofing (see Figure 21) and breathability, further expanding the range of high-performance wearable materials.

**Figure 22:**
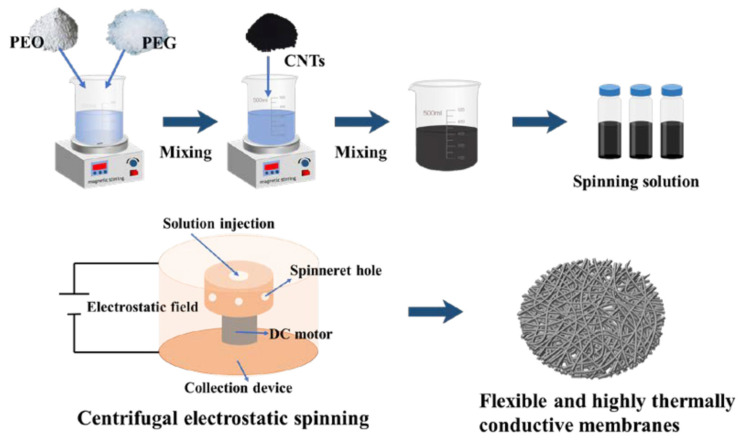
Centrifugal electrostatic spinning for the preparation of flexible and highly thermally conductive phase-change thermal storage membranes (reprinted and adapted with permission under a Creative Commons license (CC BY 4.0) from Ref. [261]).

Qiao et al. in [261] prepared PEG/PEO/CNT phase-change composite fibers via centrifugal electrostatic spinning, demonstrating excellent flexibility, thermal conductivity, and energy storage capacity (see Figure 22). These results underscore the strong potential of PCM-integrated nanofibers for flexible wearable thermal management. Collectively, these studies highlight the rapid advancement of electrospun nanofibers as multifunctional materials capable of integrating thermal regulation, antibacterial activity, breathability, and mechanical robustness. The strategic combination of lightweight synthetic fibers with breathable natural polymers further enhances comfort and performance, positioning these materials at the forefront of next generation wearable electronics and smart textile technologies.

### 6.3. Integrated Hybrid Structures for Comfort, Signal Stability, and Skin Health

The distinction between active and passive smart fibers is increasingly blurred, as many modern systems incorporate characteristics of both. Electrospinning plays a central role in this convergence by enabling the fabrication of coaxial nanostructures in which functional agents embedded in the core can interact with the environment to trigger smart behaviors such as self-healing. Unlike purely stimuli-responsive or purely conductive fibers, hybrid electrospun fibers rely on the integration of additional materials or layered assemblies to achieve complex, multi-modal responses [250]. This hybrid design philosophy forms the foundation for next-generation wearable systems that must simultaneously ensure comfort, stability, and skin compatibility. Hybrid structures incorporating shape-memory components offer unique advantages for wearable applications. Liguori et al. in [262] demonstrated this by embedding a shape-memory electrospun network within a supporting matrix, forming an internal vascular-like structure capable of dynamic deformation. Thermoresponsive nanofibers within this network undergo pronounced volume changes in aqueous environments as temperature varies, enabling materials that adapt to body conditions or external climates. Such adaptive behavior enhances wearer comfort and ensures consistent contact between the material and the skin, which is essential for stable sensing performance.

Thermoresponsive polymers further expand the functional capabilities of hybrid electrospun structures. These polymers undergo reversible transitions between coil and globule states, enabling temperature-dependent changes in fiber morphology and mechanical behavior. Liu et al. in [263] demonstrated this principle using a bilayer electrospun actuator composed of ABP-crosslinked P(NIPAM) and TPU. The structure exhibited rapid, reversible actuation between 0 °C and 40 °C, highlighting the potential of thermoresponsive nanofibers to serve as active components in wearable systems requiring dynamic fit, motion assistance, or adaptive ventilation. This work underscores how thermoresponsive fibers contribute not only to comfort but also to functional adaptability. Hybrid electrospun structures can also integrate biochemical responsiveness to support skin health. Zhang et al. in [129] developed a pH/NIR dual-sensitive electrospun membrane designed to combat bacterial infection. By encapsulating curcumin and ICG within a ZIF-8/PLA fibrous matrix and subsequently immersing the fibers in an LA-SA mixture, they created a system capable of releasing curcumin in response to two triggers: NIR-induced phase change of the PCM and the acidic environment associated with bacterial activity. This dual-stimuli mechanism demonstrates how hybrid fibers can simultaneously provide thermal responsiveness and targeted antimicrobial action, supporting both skin protection and therapeutic function.

Beyond biochemical and thermal responsiveness, hybrid structures also enable advanced multimodal sensing. Shu et al. in [264] fabricated a vertically stacked sensor capable of simultaneous temperature and pressure detection, illustrating how layered electrospun architectures can support multiple sensing pathways without signal interference. More broadly, multifunctional sensor systems can be designed either by creating a single module responsive to multiple stimuli or by integrating several single-stimulus modules into a unified structure [155]. In matrix-type systems, each sensor responds exclusively to its designated stimulus, enabling simultaneous detection of multiple environmental factors and the generation of independent electrical signals. Synchronous multifunctional sensing can be achieved through simple lamination techniques, as demonstrated in recent work [265], offering a scalable route to complex wearable sensor arrays. Together, these studies highlight how integrated hybrid electrospun structures provide a powerful platform for achieving comfort, signal stability, and skin health in wearable systems. By combining shape-memory behavior, thermoresponsive actuation, bio-chemical responsiveness, and multimodal sensing, hybrid fibers overcome the limita-tions of traditional single-function materials. This integrated approach is essential for the development of next-generation smart textiles capable of long-term, reliable inter-action with the human body.

## 7. Smart Thermoregulatory Textiles: Use Cases, Challenges, and Future Outlook

### 7.1. Use Cases

As global temperatures rise and heatwaves become more frequent, personal thermal management very relevant to prevent health risks and maintain body function, especially outdoors. Conventional cooling systems work indoors but consume large amounts of energy. Personal cooling solutions that control the body’s microclimate provide a scalable, energy-efficient alternative for everyday comfort. However, traditional fabrics lack the thermal conductivity and moisture management needed to withstand extreme or prolonged heat [246].

**Figure 23:**
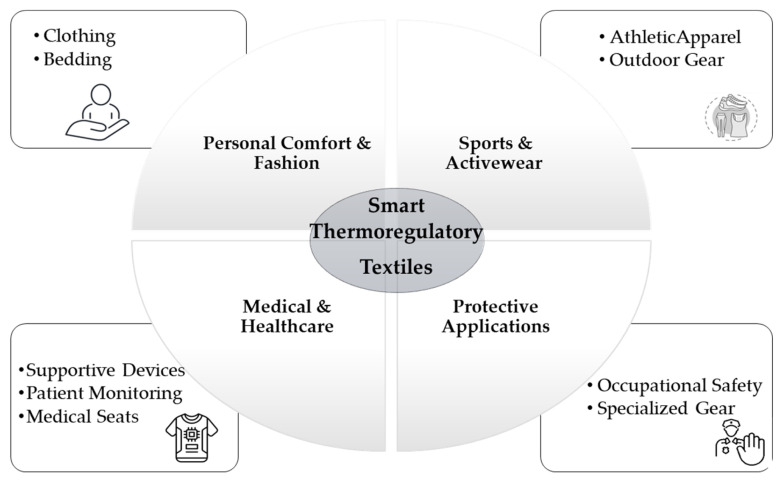
Use cases of smart thermoregulatory textiles.

Smart thermoregulatory textiles are used in a wide range of applications to manage body temperature and provide comfort, including sports and active wear, medical products, healthcare and bedding, protective clothing, medical treatments such as monitoring patient biometrics, and even spacesuits for extreme environments [220]. Conductive biomaterials-based nanomaterials for wearable sensors application areas are presented in Figure 23.

Stretchable temperature sensors can be applied across many fields, including wearable healthcare devices, monitoring the performance of mechanical and electronic systems, physiological tracking instruments, and smart packaging. They are typically built on elastic supports or flexible matrices to maintain functionality under strain. In many cases, these sensors are paired with stretchable strain sensors, enabling uses such as recording body movements, supporting therapeutic devices, and monitoring vital signs like heart rate in real time [109].

The PCMs have been applied across a broad spectrum of energy-intensive sectors, including solar energy, industrial heat-recovery systems, electrical power-peaking regulation, textiles, healthcare, liquefied natural gas processing, greenhouse agriculture, building technologies, and aerospace [254,266]. Their use is primarily concentrated in two major domains: thermal energy storage (TES)—facilitating the efficient utilization and conservation of waste heat and solar energy in industrial operations and buildings—and passive thermal regulation, where PCMs mitigate temperature fluctuations without external energy input. For these purposes, PCMs may be incorporated either as micro- or nanocapsulated systems or as form-stable PCM composites [267].

According to Sharma et al. in [268], polymer bionanocomposites are also employed in optical-fiber sensing applications. When deposited as functional coatings on optical fibers, these materials enable sensitive detection of parameters such as strain, temperature, pressure, and refractive index.

The usage of polymer bionanocomposites with properties such as flexibility, light weight, and compatibility are benefits of using them in wearable energy harvesting. These wearable technologies that are self-powered and environmental-friendly are possible to integrate into clothing, accessories, and wearable gadgets [222].

Smart thermoregulatory textiles are reshaping wearable technology by combining comfort regulation with continuous health monitoring. Electrospun biomaterials, with their high surface area, tunable porosity, and biocompatibility, provide an ideal substrate for integrating sensors into fabrics. Their nanofibrous architecture and skin-like compliance offer both comfort and functionality, making them well-suited for long-term wear. By merging passive thermoregulatory strategies with active mechanisms, these fibers enable closed-loop thermal management that adapts to physiological signals and environmental changes while maintaining breathability and safety.

Thermoregulation is central to human health and comfort, yet traditional climate control systems waste energy by heating or cooling entire spaces rather than individuals. Smart textiles, enhanced by nanotechnology and electrospinning, address this inefficiency by offering personalized thermal management while embedding biosensors for real-time monitoring. The impact of such textiles could be transformative in healthcare, elder care, athletic performance, and occupational safety, where adaptive thermal control reduces heat stress and improves well-being. Electrospun biomaterials such as polycaprolactone, silk fibroin, and cellulose derivatives are particularly promising due to their biocompatibility and ability to host functional coatings or embedded electronics [18,209].

However, challenges such as biocompatibility, durability, scalable manufacturing, and power management must be addressed before commercialization. Future progress will depend on developing autonomous, battery-minimal systems supported by advances in adaptive fabrics, bioinspired fiber designs, and sustainable manufacturing. Ultimately, electrospun smart textiles may evolve into personalized thermal homeostasis systems, redefining wearable technology as active partners in human health, comfort, and sustainability.

Smart thermoregulatory textiles built on biocompatible electrospun biomaterials have diverse applications across healthcare, performance, and sustainability. Garments and fabrics can dynamically warm or cool based on a wearer’s temperature, activity, and environmental conditions. By adjusting insulation or ventilation, they maintain comfort across varied metabolic profiles while reducing reliance on building-wide heating and cooling systems [18,209].

Integrated biosensors allow continuous tracking of vital signs such as skin temperature, hydration, sweat composition, and metabolic markers. Continuous thermoregulation aids patients with impaired autonomic control or fever management, while on-fabric sensors enable early detection of thermal stress or infection trends [18,209,269,270,271].

Thermally adaptive base layers manage heat load during exertion, prevent overheating, and provide real-time physiological feedback. Post-exercise, targeted warming supports muscle recovery and circulation, enhancing both safety and performance [209,269].

Smart uniforms and protective layers for extreme environments (construction, mining, military) deliver localized heating or cooling while monitoring thermal strain. This adaptive functionality mitigates risks of heat illness or cold injury, improving resilience and safety [18,209].

By focusing on individual thermal comfort, smart textiles reduce energy waste compared to centralized climate control systems. [3] In addition, piezoelectric electrospun textiles (e.g., PVDF-TrFE) can convert movement and temperature fluctuations into electricity, creating self-sustaining systems for powering sensors and low-power thermoregulation circuits [272,273].

### 7.2. Challenges

Electrospun materials are lightweight, biocompatible, and versatile, enabling their integration into a wide range of applications. Although electrospinning technology shows considerable promise for healthcare-related sensing and has potential for broad implementation in daily life, several challenges remain. These include the complexity of fabricating advanced sensor materials, high production costs, and the inherently limited mechanical robustness of many electrospun structures, which compromises long-term operational stability and restricts large-scale deployment. Furthermore, improvements in sensitivity and response time are still required. As electrospinning continues to incorporate increasingly sophisticated material systems, its utility in biosensing applications is expected to expand. Future progress in this field will depend on addressing issues related to mass production and cost efficiency, necessitating multidisciplinary collaboration across areas such as machinery, computer science, and biomedical engineering [274].

**Figure 24:**
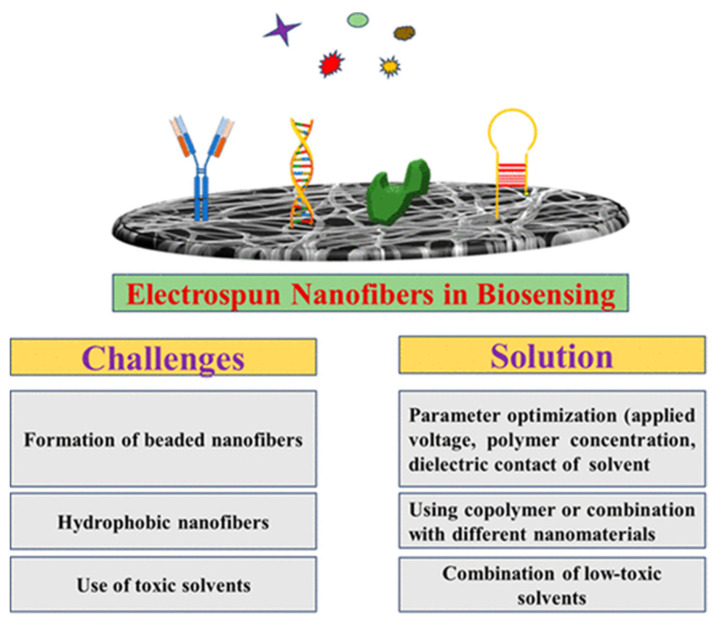
Challenges and solutions of electrospun nanofibers in biosensor systems (reprinted and adapted with permission under a Creative Commons license (CC BY 4.0) from Ref. [276]).

Technical and material challenges, such as inhomogeneity in nanofiber mats, variability in fiber alignment and thickness during electrospinning can lead to inconsistent sensor performance. In order to overcome these challenges, the process is optimized. Fine-tuning electrospinning parameters such as polymer viscosity, electric field strength, and solution properties can improve fiber uniformity and reduce the occurrence of issues like needle clogging [275,276]. Limitations and solutions of electrospun nanofibers in biosensor systems are presented in Figure 24.

Another challenge is material selection and compatibility, that means finding polymers that are simultaneously flexible, breathable, biocompatible, and responsive to temperature changes are doubtful. Developing thinner, more flexible electrodes that can functionalize without gel electrolytes is a potential alternative for long-term, comfortable monitoring [277].

Electrospun wearable sensors with thermoregulation property have specific issues, such as precision in temperature sensing. Achieving accurate and stable temperature readings in dynamic environments is difficult due to external interference like humidity and sweat. Most current systems focus on passive thermoregulation (e.g., insulation or breathability). Integrating active control (e.g., heating/cooling) requires complex energy management and miniaturized components. Other issues are integration and functionality. Sensor fusion (assembling) and of course, data accuracy.

Lightweight, flexible power sources are needed to support continuous operation without bulky batteries. Also, wearables must withstand repeated use and washing without degrading-electrospun fibers often struggle with mechanical robustness. These materials should stand out such properties as durability and washability.

As usually, one of the biggest challenges is large-scale production. Electrospinning is difficult to scale for mass production, and variations in parameters like voltage, temperature, and humidity can lead to inconsistent fiber diameters and morphologies, affecting sensor performance.

Various research groups have proposed strategies for integrating electrospun nanofibers or nanofiber mats into smart textiles. One of them is based on the production of nanofiber yarns [278,279]. Being a relatively new field, nanotextile research still faces several challenges. Nanotextiles have already demonstrated strong performance and compatibility in biomedical applications—including tissue engineering, wound healing, and drug delivery—where biocompatibility and biodegradability are essential. However, for wearable applications such as clothing, additional requirements arise: the materials must be washable, breathable, and scalable for large-volume production. Achieving these properties simultaneously remains difficult, and further advancements are needed to overcome these limitations [278].

The development of hybrid systems faces significant challenges and incompatibilities, including data processing, calibrations and use comfort [221]. Also, recycling of these advanced materials presents unique challenges due to their complex, multi-component structures. Many electrospun membranes combine polymers, PCMs, nanoparticles, and bioactive compounds within a single fiber architecture. This mixing complicates mechanical or chemical separation at the end of life.

Wearable devices can provide timely, user-friendly, non- or minimally invasive, and continuous monitoring of human health [260].

In total, despite significant progress, several challenges must be addressed before biocompatible electrospun smart textiles can achieve widespread commercialization. Biocompatibility and skin safety remain critical, as continuous contact with human skin requires hypoallergenic, breathable, and non-cytotoxic fibers, yet the inclusion of conductive fillers, phase-change materials, or photothermal coatings can introduce potential irritants, creating a trade-off between performance and dermatological safety. Durability and washability are also major hurdles, since repeated mechanical stress, sweat exposure, and laundering can degrade fiber integrity and compromise sensor accuracy. Power supply and management limit the operation of active heating or closed-loop systems, while motion artifacts, perspiration, and variable fit challenge signal stability and data reliability. Manufacturing scalability and cost further constrain adoption, as electrospun and nanocomposite fibers require precise process control, specialized equipment, and standardized protocols that are difficult to implement at industrial scale. Finally, the lack of uniform standards, interoperability, and ergonomic optimization can delay regulatory approval, reduce system compatibility, and impact user comfort, directly affecting consumer acceptance. Collectively, these interrelated challenges highlight the need for an integrated approach that combines advanced material design, reliable electronics, scalable manufacturing, and user-centered ergonomics to deliver safe, durable, multifunctional, and commercially viable smart textiles. The summary of these challenges is presented in Table 6.

Life cycle assessment (LCA) remains a significant challenge in the development of biocompatible electrospun biomaterials for thermoregulating wearable sensors. Although bio-based and biodegradable polymers—including silk fibroin, cellulose, chitosan, alginates, polylactic acid (PLA), and polycaprolactone (PCL)—are frequently promoted as sustainable alternatives to petroleum-derived materials, their environmental performance must be evaluated across the full cradle-to-grave life cycle using standardized LCA methodologies (ISO 14040 [281] and ISO 14044 [282]) rather than inferred solely from biocompatibility or biodegradability [115,120,121,130,137,138,142,150,153,236,283]. Electrospinning is often associated with high energy consumption and the use of volatile organic solvents, which can substantially contribute to global warming potential and human toxicity indicators, particularly when solvent recovery and energy sourcing are not optimized [236,248,284]. Furthermore, the absence of harmonized functional units and system boundaries in existing LCAs of smart and functional textiles hampers meaningful comparison between studies [236]. For wearable sensor applications, the use phase is particularly critical, as limited durability, wash resistance, and short operational lifetimes of electrospun smart textiles can significantly increase environmental impacts per functional unit [156,175,209,280]. These uncertainties highlight the need for LCA frameworks specifically tailored to functional and smart textile systems rather than conventional apparel products.

Recycling and end-of-life management pose additional barriers to the sustainability of electrospun smart textiles. While electrospun biomaterials such as PLA, cellulose derivatives, and silk fibroin offer potential biodegradability [115,120,121,150,153,283], their integration with conductive fillers, thermoresponsive coatings, phase-change materials, and electronic components results in highly heterogeneous, multi-material structures [156,158,165,192,209]. This complexity limits the feasibility of conventional mechanical recycling and may inhibit biodegradation under real disposal conditions, especially when chemical modification, crosslinking, or inorganic nanofillers are employed to enhance durability and functionality [119,128,136,142,163]. In particular, the incorporation of metallic interconnects, carbon-based nanomaterials, or conductive polymers introduces e-waste considerations, often diverting smart textiles toward incineration or landfill rather than circular recovery routes [156,208,211,236]. As a result, the environmental advantages of biocompatible electrospun substrates may be partially negated at the end-of-life stage.

From a circular economy perspective, a key challenge lies in aligning material selection with design-for-recycling and design-for-disassembly principles. Emerging strategies such as modular sensor architectures, detachable electronic components, and closed-loop recycling approaches—including chemical recycling and pyrolysis-based upcycling of textile waste into functional carbon materials—have shown promise at the laboratory scale [156,213,236]. However, these approaches face challenges related to scalability, economic feasibility, and regulatory compliance, particularly for complex thermoregulating systems incorporating phase-change materials and hybrid polymer networks [192,237]. Integrating LCA-driven decision-making at the early design stage is therefore essential to ensure that improvements in sensing performance and thermoregulation do not come at the expense of increased environmental burden [156,236]. Addressing these challenges will be critical for advancing electrospun smart textiles from experimental prototypes toward truly sustainable, next-generation wearable technologies.

### 7.3. Future Outlook

The future of biocompatible electrospun biomaterials in smart thermoregulatory textiles is promising. Seamless integration of skin-mounted temperature and strain sensors with distributed Joule heating and infrared-tuned layers will enable autonomous thermal homeostasis driven directly by physiological signals [18,209]. Advancements in piezoelectric and triboelectric electrospun fibers, along with hybrid energy harvesters, will power low-duty-cycle sensors and microheaters, reducing reliance on bulky batteries and enhancing wearability [272,273]. Electrically or mechanically tunable infrared emissivity, inspired by metamaterials and enabled by stretchable conductors, will allow textiles to switch dynamically between cooling and heat retention on demand [18,209]. Hierarchical porosity, capillary pathways, and moisture-responsive polymers will mimic skin’s natural thermoregulation, providing comfort without heavy energy input and strengthening biocompatibility during long-term wear [18,209]. Next-generation fabrics will combine thermoregulation with biosensing, energy harvesting, and therapeutic delivery, creating truly multifunctional platforms [18]. Machine learning algorithms will enable textiles to adapt dynamically to individual physiology and environmental conditions, optimizing comfort and health outcomes [209]. Advances in eco-friendly biomaterials, solvent systems, recyclable biopolymers, and standardized fiber metrics will reduce environmental impact, support circular economy models, and lower regulatory barriers [18,280]. Smart textiles are expected to play a role in managing chronic conditions such as diabetes, cardiovascular disease, and rehabilitation, through continuous monitoring and responsive feedback. Longitudinal trials across diverse populations, transparent data practices, and clinically relevant endpoints (e.g., heat stress prevention, fever detection) will be critical for healthcare integration [209,271]. Seamless integration with wearable devices, smartphones, and smart environments will allow textiles to function as interconnected nodes in digital health and comfort ecosystems [269,270].

Research is ongoing to develop new techniques like electrospinning-electrospraying to improve the connection between electrospun fibers and enhance thermal conductivity. Exploring new combinations of polymers and conductive fillers, along with optimized processing parameters, continues to be an area of active research.

Future textile thermal controllers will integrate advanced sensing and control mechanisms, such as distributed or infrared temperature sensors and indirect heater performance monitoring. They will also consider factors like user behavior, wear duration, and battery status to dynamically adjust heat output, moving beyond fixed temperature settings to meet real-time needs and preferences.

Advanced electrospinning methodologies—such as coaxial electrospinning, aligned electrospinning, yarn electrospinning, and roll-to-roll fabrication—highlight the significant scalability and versatility of this technology for producing next-generation nanomaterials. These approaches enable precise structural control, continuous manufacturing, and integration into large-area formats, underscoring electrospinning’s potential for advanced material engineering [284].

Flexible shape-stabilized composite PCMs provide clear advantages over conventional PCMs, offering flexibility, lightweight design, intelligence, and wearability. Research is needed to balance flexibility with thermal storage capacity and improve thermal conductivity for better regulation. There is strong potential for wearable, self-repairing energy storage devices and integration with advanced functions. For practical use in harsh conditions, stable flexibility and durability must be ensured, supported by improved characterization methods. Developing low-cost, large-scale production technologies remains a key challenge, while emerging applications such as thermoelectric conversion, nanogenerators, and electromagnetic shielding highlight the wide potential of flexible PCMs [231,285].

Future works focuses on balancing flexibility vs. thermal storage capacity in hybrid PCMs [286]. Also, developing low-cost, scalable production methods for large-scale textile applications [229]. Advancing characterization techniques of materials (nanoindentation, pre-stretching tests) to better understand flexibility mechanisms [228]. Exploring self-repairing and multifunctional devices that integrate energy storage with thermal regulation [192].

Electrospinning represents an effective approach for producing thin biopolymer films, offering substantial flexibility in tailoring nanofibrous structures to improve overall device performance. In contrast, additive manufacturing—particularly 3D printing—enables a significantly higher degree of structural control, allowing the fabrication of complex geometries and compatibility with a wide range of complementary processing techniques, including extrusion, grinding, rolling, and ball milling. Looking ahead, the advancement of functionalized biopolymers for energy-storage applications should emphasize the development of materials capable of complete degradation after a defined service life, thereby minimizing waste generation. Integrating components derived from functionalized biopolymers into electronic devices offers several advantages, such as non-toxicity, straightforward synthesis, ease of device fabrication, and compatibility with functional fillers [216].

Nanofibrous constructs frequently exhibit insufficient mechanical robustness to accurately replicate the functional behavior of structurally complex tissues. Consequently, the ability to engineer geometries with greater precision and architectural fidelity remains a central challenge. Within this framework, additive manufacturing approaches—particularly 3D printing—offer a promising route for creating customized structures with tightly controlled architectures and significantly enhanced mechanical performance [8]. Electrospinning can create the high-surface-area nanofiber mats that act as the core sensing component, while 3D printing can be used to build a flexible, comfortable, and durable substrate or housing for the sensors.

Hybrid manufacturing: Some advanced techniques even combine the two processes, using 3D printing to create a scaffold and then electrospinning onto it to add the functional nanofibers [73].

Future research should also prioritize the integration of electrospinning with artificial intelligence (AI), leveraging AI-driven optimization of processing parameters and environmental conditions to improve material consistency and performance. In parallel, the application of materials-genome technologies offer a powerful route to accelerate the discovery of new compositions, reveal structure–property relationships, enhance production efficiency, and address key industrialization barriers. Together, these approaches have the potential to significantly advance the scalability and functional capabilities of electrospun materials [274].

In essence, natural-synthetic electrospun hybrids represent a promising pathway toward sustainable, high-performance thermoregulating textiles and devices, merging eco-friendly materials with advanced engineering for real-world applications.

## 8. Conclusions

Biocompatible electrospun biomaterials provide a versatile and promising foundation for next-generation smart thermoregulatory textiles. Their high surface area, tunable porosity, nanofibrous architecture, and skin-like compliance make them uniquely suited for embedding wearable sensors and thermoregulatory functions while maintaining comfort, breathability, and biocompatibility. By combining passive strategies such as phase-change materials and infrared emissivity tuning with active mechanisms like Joule heating and photothermal conversion, electrospun fibers enable closed-loop thermal management that adapts to physiological signals and environmental changes.

The impact of these textiles could be transformative across healthcare, elder care, athletic performance, and occupational safety. By coupling sensing with adaptive thermal control, they can mitigate heat stress, enhance recovery, and improve overall well-being. Their potential extends further into continuous health monitoring, where integration with biosensors supports real-time detection of thermal stress, hydration levels, and metabolic changes.

Despite promising laboratory demonstrations, challenges remain. Long-term biocompatibility, durability under washing and wear, scalable manufacturing, and reliable power management are persistent barriers to commercialization. Addressing these issues will require interdisciplinary collaboration across materials science, textile engineering, and biomedical design, alongside the development of standards and certification pathways.

Looking ahead, progress will depend on closed-loop, battery-minimal systems that autonomously regulate microclimates based on physiological signals. Advances in adaptive emissivity fabrics, bioinspired fiber architectures, and scalable green manufacturing will accelerate translation into real-world applications. The convergence of multifunctional design, AI-driven personalization, sustainable production, and IoT connectivity will further transform smart textiles into holistic platforms for comfort, healthcare, and energy efficiency.

Ultimately, biocompatible electrospun smart textiles could evolve into personalized thermal homeostasis systems, seamlessly merging sensing, actuation, and energy harvesting. Such systems would redefine wearable technology—not merely as passive monitors, but as active partners in human health, comfort, and sustainability, positioned as integral components of future digital health and sustainable living ecosystems.

## Figures and Tables

**Table 1:** Comparison of Solution Electrospinning vs. Melt Electrospinning.

Parameter	Solution Electrospinning	Melt Electrospinning
Processing method	Polymer dissolved in solvent	Molten polymer without solvent
Fiber diameter	Nanometer to micrometer scale due to strong jet thinning from solvent evaporation	Typically micrometer-scale because no mass loss occurs during jet travel
Material compatibility	Extremely broad polymer range including biopolymers, conductive polymers, and composites	Limited to thermoplastic, thermally stable polymers
Temperature sensitivity	Low processing temperatures allow incorporation of enzymes, antibodies, and bioactives	High temperatures prevent use of thermally sensitive additives
Environmental impact	Solvent use introduces toxicity and VOC concerns	Solvent-free and environmentally friendly
Mechanical strength	Dependent on solvent removal and polymer crystallinity	Generally higher strength due to dense melt-state polymer chains
Energy consumption	Lower energy demand (no melting required)	Higher energy demand due to heating above melting point
Scalability	Widely scalable but requires solvent recovery systems	Highly scalable for industrial production
Fiber placement precision	Limited due to jet whipping	High precision possible via melt electrowriting
Suitability for smart textiles	Ideal for ultrafine sensing layers and bioactive coatings	Ideal for durable structural layers and patterned conductive paths

**Table 2:** Typical solvents utilised in solution nanospinnning.

Fiber Forming Polymer	Solvents		Key Fiber Properties
Biopolymer	gelatin	acetic acid, formic acid, water/ethanol mixture	Biodegradable, flexible, high surface area; fiber uniformity sensitive to solvent composition
	collagen	acetic acid, 1,1,1,3,3,3-hexafluoro-2-propanol (HFIP)	Biocompatible, protein-based, tunable porosity
	silk fibroin	formic acid, HFIP, water	Strong, elastic, tunable fiber diameter
	chitosan	acetic acid, trifluoroacetic acid (TFA), acetic acid	Antimicrobial, hydrophilic, fiber diameter sensitive to concentration
	cellulose acetate	acetic acid, acetone, DMF, DCM,	Biodegradable, thermally stable, uniform fibers
Synthetic polymer	poly(vinyl alcohol) (PVA)	water, water/DMSO mixture, water/ethanol mixture	Hydrophilic, water-processable, moderate mechanical strength
	poly(ε-caprolactone) (PCL)	acetone, chloroform, DMF, DCM/methanol mixture	Flexible, mechanically robust, slow degradation
	polyurethane (PU)	DMF, THF, DMF/acetone mixture	Stretchable, elastic, chemical resistant
	poly(lactic acid) (PLA)	acetone, chloroform, DMF, DCM	Biodegradable, moderate strength, tunable porosity
	polyacrylonitrile (PAN)	DMF, DMSO	High thermal stability, electrically conductive with fillers

**Table 3:** Biomaterials conductivity enhancing additives for electrospinning.

Additive Type	Examples	Functional Role in Wearable Textiles	Reference
conductive polymer	PEDOT: PSS, polyaniline (PANI), polypyrrole (PPy)	Provide electrical conductivity, enable flexible circuits, support strain/pressure sensing, facilitate Joule heating for thermal management.	[34,90]
carbon-based nanomaterial	graphene, graphene oxide (GO), reduced GO, carbon nanotubes (CNTs), carbon black, etc.	Enhance electrical and thermal conductivity, improve mechanical strength, enable electrochemical and optical sensing, assist in heat dissipation.	[102,103,104]
metal nanoparticles (NPs)	silver (AgNPs), gold (AuNPs), platinum (PtNPs), copper (CuNPs)	Impart high electrical conductivity, catalytic activity, antibacterial properties, plasmonic/photothermal effects for adaptive heating and sensing.	[96]
inorganic MXenes	Ti_3_C_2_Tx and other 2D carbides/nitrides	Provide high electrical and thermal conductivity, electrochemical activity, mechanical reinforcement, and multifunctional sensing capabilities.	[25,105,106]

**Table 4:** Comparison of natural and synthetic electrospun polymers.

Polymers	Type of Polymer	Advantages	Limitations	Reference
Natural electrospun polymer	Silk fibroin, cellulose, chitosan, gelatin	High biocompatibility and comfort; Naturally breathable and moisture-absorbing; Sustainable and biodegradable.	Weaker, sensitive to moisture Less effective in long-term thermal regulation without modification	[112,113,115,117,156]
Synthetic electrospun Polymers	Polyurethane, polycaprolactone, polyethylene oxide, polyacrylonitrile	Durability and tunability in fiber diameter, porosity, and crystallinity. Can embed PCMs, conductive fillers, or dyes for precise thermal control. Better mechanical strength and washability.	Lower biocompatibility; often hydrophobic. Environmental concerns (non-biodegradable, microplastic release). Require surface modification for comfort and moisture management	[18,143,157]

**Table 5:** Nanofibers reinforced with different PCM and additives using electrospinning technique.

Method	Polymer/PCM	Additive	Functionalization	Reference
Uniaxial/Coaxial electrospinning	PCL/PEG	-	Melting enthalpy: 39.5 × 103 J/kg; Thermal conductivity: 0.1662 W/mK/Smart fabrics, biosensors	[236]
	PCL/PEG	Curcumin	Biomedical (drug release and antioxidant activity)	[237]
	PCL/PEG	-	The thermal conductivity of PCL@PEG70 could go up to 0.1662 W/mK, increasing by 49.5% compared with that of PCL.	[238]
	PU/PEG	SiO_2_	Increased visible and near-infrared light reflectance, superwetting, photothermal regulation	[239]
	PU/PEG	Si_3_N_4_	High thermal conductivity (4504 mW/m K); Increased solar reflectance (91 %), high infrared emissivity (92 %),	[240]
	PU and CNF/Stearic acid	Mica mineral	Enhanced electrical resistivity and UV] reflectance	[241]
	PVA/PEG	AgNO_3_; TiO_2_	Enhanced thermal properties (decrease in supercooling effect)	[242]
	PVA/Lauric acid (LA)/(PCM)	MWCNTs/ZnO particles coated with a green PDMS layer	Thermal conductivity of PVA -(0.334 W·m^–1^·K^–1^); HPCF −0.665 W·m^–1^·K^–5^; excellent UV-protection	[197]
	PVA/PEG	-	Heat enthalpies of PEG/PVA were 78.806 J/g; Excellent thermal stability; Good thermal regulation.	[243]
	PVA/PEG	CNT	Increased mechanical strength and thermal conductivity (64.92 mW/m·K, 40.4 % increase)	[244]
	PVA/Paraffin	Polypyrrole (PPy) coating; Silver nanowires (AgNWs)	Increased thermal conduction pathways, photothermal conversion, electrical conductivity (0.148 S/m), piezoresistive response range (440.6 kPa)	[210]
	PVP/PEG	GO	Improved thermal conductivity (566.8 mW/m.K)	[245]

**Table 6:** Key challenges hindering the commercialization of biocompatible electrospun smart thermoregulatory textiles.

Challenge	Description	Impact on Commercialization	Reference
Biocompatibility and skin safety	Long-term wear demands biomaterials that are safe, hypoallergenic, breathable, and non-cytotoxic. Balancing performance additives (e.g., conductive or photothermal coatings) with dermatological safety remains nontrivial, as continuous skin contact requires stable finishes that do not cause irritation or adverse reactions.	Limits adoption in healthcare and elder care if safety concerns are not fully addressed.	[18,209,269]
Durability and washability	Smart textiles must withstand repeated mechanical stress, bending, sweat exposure, abrasion, and laundering without degradation of thermal or electrical performance. Maintaining sensor accuracy and fiber integrity under these conditions is a persistent hurdle for nanomaterial-laden and electrospun systems.	Reduces product lifespan and consumer confidence in everyday use.	[18,270,273]
Power supply and management	Integration of flexible, lightweight, and sustainable energy sources remains a bottleneck. Active heating and closed-loop control require reliable energy delivery, yet energy-harvesting fibers often provide inconsistent output. Efficient storage and integration solutions are needed to support all-day wear.	Restricts continuous operation of sensors and active heating/cooling systems.	[18]
Signal stability and data reliability	Ensuring accurate sensor readings under motion, perspiration, and environmental fluctuations is essential. Motion artifacts, sweat interference, and variable fit can compromise data quality, requiring careful co-design of materials and electronics.	Weakens trust in health monitoring and performance feedback applications.	[271,273]
Manufacturing scalability and cost	Scaling electrospun and nanocomposite fibers to roll-to-roll production with consistent quality control raises costs and variability. Supply chain limitations and lack of standardization hinder broader adoption, while commercialization requires reproducible, cost-effective manufacturing.	Slows industrial adoption and increases barriers to mass-market entry.	[270,273,280]
Standards and interoperability	The absence of uniform testing methods (e.g., thermal emissivity, comfort metrics, bio-safety), certification pathways, and integration protocols with electronics delays regulatory approval and market entry.	Delays regulatory approval and hinders integration into broader wearable ecosystems.	[280]
User comfort and experience	Designing textiles that provide accurate on-body temperature sensing across diverse fits and activities, while avoiding bulk, hotspots, or discomfort, requires careful integration of materials, electronics, and ergonomic design.	Impacts consumer acceptance and usability in daily wear.	[18,209]

## Data Availability

No new data were created or analyzed in this study. Data sharing is not applicable to this article.
